# Counterfactual modeling isolates sand mining impacts, revealing it as a key driver of Mekong Delta destabilization

**DOI:** 10.1126/sciadv.aef0558

**Published:** 2026-07-08

**Authors:** Sonu Kumar, Edward Park, Dung Duc Tran, Thanh Quoc Vo, Karl Kästner, Sameh Kantoush, Doan Van Binh, Jiachun Huang, Jingyu Wang, Adam D. Switzer

**Affiliations:** ^1^ National Institute of Education, Nanyang Technological University, Singapore, Singapore.; ^2^ Earth Observatory of Singapore, Nanyang Technological University, Singapore, Singapore.; ^3^ Asian School of the Environment, Nanyang Technological University, Singapore, Singapore.; ^4^ Institute for Environment and Resources, Vietnam National University Ho Chi Minh City, Ho Chi Minh City, Vietnam.; ^5^ College of Environment and Natural Resources, Can Tho University, Can Tho, Vietnam.; ^6^ Brandenburgische Technische Universität Cottbus-Senftenberg, Brandenburg, Germany.; ^7^ Disaster Prevention Research Institute (DPRI), Kyoto University, Kyoto, Japan.; ^8^ Vietnamese-German University, Ho Chi Minh City, Vietnam.

## Abstract

Sand mining is a poorly quantified threat to river deltas because its impacts are often confounded with those of dams and climate change. Here, we isolate its effects in the Vietnamese Mekong Delta, a global sand mining hot spot, using a long-term numerical model and observed mining data. Results show that mining exceeds natural sediment supply by 6 to 15 times, causing riverbed erosion across ~65% of channels, with mean incision rates of ~0.10 meters per year (~25 to 30% of total incision driven by all drivers). This channel deepening alters flow, reduces sediment transport, and enhances saltwater intrusion. Sand mining alone contributes ~16 to 30% of the annual salinity increase, extending intrusion up to ~1.5 kilometers further inland during the dry season. These changes demonstrate that sand mining is a major, previously underquantified driver of delta instability, affecting river morphology, flow, sediment, and salinity. This study provides a framework to quantify these impacts and support better management of sand extraction in vulnerable deltas worldwide.

## Teaser

Sand mining drives ~25 to 30% of total channel erosion and ~16 to 30% of salinity increase in the Vietnamese Mekong Delta.

## INTRODUCTION

**Fig. 1.**
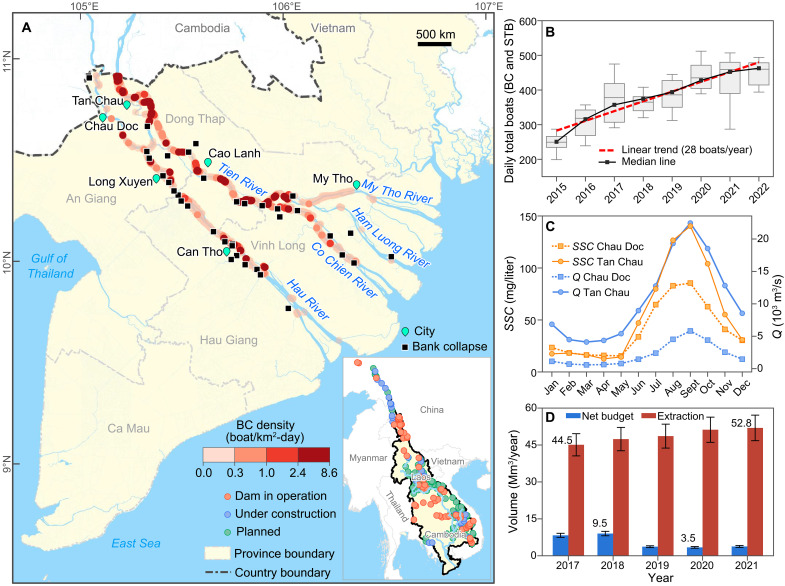
Study domain, sediment monitoring, and sand mining pressure in the VMD. (**A**) Spatial distribution of sand mining pressure and geomorphic instability across the VMD. Red circles show the density of barge-with-crane (BC) sand mining boats (boat/km^2^-day) derived from satellite observations (*11*), black squares indicate reported bank-collapse locations, and colored circles in the inset map show the status of upstream dams (operational, under construction, and planned) across the Mekong Basin. Major distributary channels and upstream boundary stations (Tan Chau and Chau Doc) are labeled. (**B**) Interannual trend in total sand mining boats [BC and sand transport boats (STB)] from 2015 to 2022, with boxplots showing annual variability and the fitted linear trend. (**C**) Mean monthly suspended sediment concentration (*SSC*; orange) and discharge (*Q*; blue) at the Tan Chau and Chau Doc upstream stations, showing the seasonal hydro-sediment regime used to define model boundary conditions. (**D**) Comparison between annual net sediment budget (natural sediment supply minus distributary outflow; blue bars) and reported sand extraction volumes (red bars) for 2017 to 2021, with error bars indicating ±10% uncertainty.

Global demand for sand has increased rapidly in recent decades, placing growing pressure on river deltas as key sources of high-quality sediment (*1*, *2*). These systems are already vulnerable to interacting environmental pressures, including climate change, land-use change, sea level rise, land subsidence, and sediment trapping by upstream dams (*3*, *4*), making them highly sensitive to additional pressure from excessive sand mining activities (*5*–*8*). The Vietnamese Mekong Delta (VMD) (Fig. 1, A to C) is a prime example of this crisis and provides an ideal testbed for this analysis because of its high extraction intensity, geomorphic sensitivity, and data availability (*9*). It has become a major sand mining hot spot, where annual extraction of 40 to 50 Mm^3^/year greatly exceeds natural replenishment (*10*, *11*). This intensive dredging accelerates channel incision and increases the risk of bank instability, contributing to widespread riverbank collapse and loss of land and infrastructure (*12*). Beyond geomorphic impacts, the consequences also extend to subsurface processes, as alterations in the hydraulic connection between rivers and aquifers can destabilize groundwater systems, reduce freshwater availability, and increase vulnerability to salinization and land subsidence (*13*, *14*). These effects have profound socioeconomic consequences for millions of inhabitants and collectively illustrate how deltas (*15*), which are already fragile under multiple environmental pressures, face existential, compounded risks when unsustainable sand mining is added (*16*). These combined pressures highlight the urgency of developing process-based approaches to isolate and quantify the impacts of sand mining in delta systems.

Research in the VMD has used both observational and modeling approaches to understand the impacts of sand mining. Observational studies have systematically documented changes in river geometry over time and quantified substantial shifts in the delta’s morphology through field surveys and multitemporal satellite imagery (*17*–*22*). These studies establish strong links between sand mining intensity and geomorphic consequences such as riverbank instability and channel incision. Beyond physical changes, research has also documented altered sediment transport pathways, degraded water quality, and biodiversity impacts associated with sand mining (*23*). To investigate these complex processes more dynamically, researchers have developed and applied a variety of modeling approaches. Hydrodynamic and morphodynamic models including TELEMAC-2D, Delft3D, and MIKE21 have been applied to simulate sediment dynamics and forecast potential system changes under various sand mining scenarios (*24*–*26*). These modeling efforts provide a framework for integrating sand mining into environmental impact assessments and developing sustainable management strategies. Despite these valuable contributions, a fundamental methodological challenge remains, to our knowledge, across both observational and modeling studies: the inability to isolate the specific impact of sand mining from the compounded effects of other anthropogenic and climatic pressures.

Observational studies, while documenting correlations, inherently capture cumulative changes driven by upstream dam sediment trapping, climate-induced sea level rise, land subsidence, and mining activities (*27*). Without isolating the sand mining signal, it remains difficult to determine its proportional contribution to key issues such as salinity intrusion or bank collapse, which is essential for targeted policy and mitigation strategies (*27*–*29*). A second limitation is that existing modeling studies rely on simplified or hypothetical assumptions, often using sensitivity analyses with assumed sand mining rates and locations due to the absence of spatially explicit dredging data (*26*, *30*). This approach fails to represent the true spatial heterogeneity and intensity of real-world extraction, leading to uncertainty in model predictions and limiting their practical value for regulatory applications. Third, there remains a lack of holistic, long-term, and fully integrated modeling frameworks. Previous studies are often constrained to short-term simulations or rely on morphological acceleration factors to approximate long-term change (*31*). Critically, key processes, including hydrodynamics, sediment transport, morphodynamics, and salinity intrusion, are frequently simulated in isolation or with static assumptions. This limits the representation of essential feedback mechanisms, such as interactions between channel incision, sediment flux, and bank erosion (*32*). Consequently, existing approaches are unable to robustly quantify the long-term, system-wide impacts of sand mining, leaving its contribution to delta destabilization poorly constrained and representing a critical gap for sustainable management.

We address these gaps here by developing a comprehensive process-based modeling framework to isolate and quantify the systemic impacts of sand mining in the VMD. Our approach integrates three key advances to enable robust, delta-scale, process-based attribution of environmental change to sand mining. First, we use a counterfactual methodology that isolates sand mining impacts by simulating the system with and without extraction under identical boundary conditions, thereby separating its signal from other drivers. Second, we incorporate a spatially explicit dataset of observed sand mining volumes, ensuring that simulations reflect present-day extraction patterns rather than hypothetical scenarios. Third, we implement a fully integrated Delft3D-FM framework that couples hydrodynamics, sediment transport, morphodynamics, and salinity intrusion over a multiyear period without morphological acceleration. We further resolve how mining alters tidal-river dynamics, a key but poorly quantified process in delta systems. Together, this approach captures the cascading effects of sediment removal on geomorphic and hydrodynamic processes and their implications for freshwater security. By resolving these coupled dynamics under present-day extraction conditions, this framework provides a robust, process-based basis for attributing mining-driven changes and identifying its role as a key driver of delta destabilization.

## RESULTS

### Integrated model performance for river dynamics

The hydrodynamic model exhibited strong performance in reproducing discharge and water levels across the VMD, with Willmott Skill Scores (WSSs) of 0.94 for water level and 0.97 for discharge, indicating reliable representation of tidal forcing and seasonal river dynamics. Simulated discharge at the two stations with available observations, Can Tho and My Thuan, also showed high agreement [coefficient of determination (*R*^2^) ≈ 0.88], suggesting that large-scale flow partitioning between the Tien and Hau systems was well captured. The coupled hydrodynamic-salinity model reproduced seasonal salinity intrusion, capturing upstream saline penetration during low-flow conditions, with daily-scale skill comparable to hydrodynamic metrics (WSS ≈ 0.91). Agreement improved with temporal aggregation, indicating that basin-scale controls on salinity dynamics were well resolved. The sediment transport module reproduced suspended sediment variability at the two observation stations with moderate to good skill (WSS ≈ 0.66 to 0.87 across temporal scales), resolving seasonal variability despite limited spatial coverage and greater uncertainty in event-scale SSC peaks (*24*). Morphodynamic performance was further supported by validation against bathymetric data, with domain-scale agreement consistent with established benchmarks (WSS ≈ 0.81), indicating that the model captured broad channel morphology and sediment redistribution patterns, although local cross-sectional agreement varied spatially. Detailed performance metrics, station-level comparisons, and extended validation analyses are provided in Supplementary Text 1 and figs. S1 to S6. Overall, model performance was broadly consistent with, and in some cases exceeded, established thresholds for large-river delta systems (*33*–*35*), providing an appropriate basis for simulation of river dynamics. All scenarios were simulated using identical model configurations and forcing conditions. As a result, any systematic model biases are consistent across simulations, and the analysis focuses on relative differences between mining and no-mining conditions, thereby minimizing the influence of absolute model uncertainties.

### Basin-scale sediment budget and systemic reduction of sediment storage

Basin-scale sediment budgets constrain long-term sand mining impacts in the VMD. Our analysis revealed a persistent sediment deficit, with extraction far exceeding upstream supply. Net sediment supply declined from ~9.5 Mm^3^/year in 2018 to ~3.5 Mm^3^/year in 2020 (Fig. 1D). This decline was driven by increased sediment trapping behind dams commissioned after 2017, reducing sediment delivery to the delta [table S1; (*36*)]. In contrast, reported sand mining volumes remained high, increasing from ~44.5 Mm^3^/year to 52.8 Mm^3^/year in 2021, with a 5-year mean of ~48 Mm^3^/year (*11*). This created a widening imbalance, with extraction exceeding natural replenishment by factors of 6 to 15 annually. This imbalance exceeded the global average, where extraction typically surpasses supply by about a factor of 2, highlighting the intensity and unsustainable nature of sediment mining in the VMD (*37*). In contrast to climate-sensitive sediment supply, extraction remained steady, reflecting sustained dredging driven by urban growth and construction demand (*19*). Cumulatively over the 5-year period, the total sediment deficit exceeded ~250 Mm^3^, resulting in a basin-wide reduction in channel sediment storage. Comparison of simulated bed-sediment layers (fig. S7, A and B) showed extensive depletion across deltaic channels. Under the no-mining scenario, thicker and more continuous sediment layers were maintained, particularly along distributary thalwegs. In contrast, the mining scenario exhibited widespread loss of near-bed sediment storage, especially upstream of bifurcations and near extraction hot spots such as My Thuan. The histogram (fig. S7E) showed a shift toward lower sediment-thickness classes: The thinnest class (<14-m thickness) expanded by ~67%, while the dominant midthickness class (15.0 to 16.0 m) declined by 8%. Thicker classes (>17.0 m) decreased by ~10%, whereas the thickest zones (>18 m), representing ~32% of the area and concentrated in downstream reaches, remained nearly stable (−0.8%). This pattern suggests that sand extraction preferentially depletes sediment storage in upstream and midchannel reaches, while downstream thick-sediment zones are partly buffered by sustained deposition and therefore show smaller relative change. Overall, the redistribution toward thinner sediment classes indicates progressive sediment depletion under sustained sand extraction, implying reduced channel sediment storage capacity and potentially lower vertical resilience of the delta bed.

### Morphological responses to sand mining

#### Spatial patterns of erosion and deposition

**Fig. 2.**
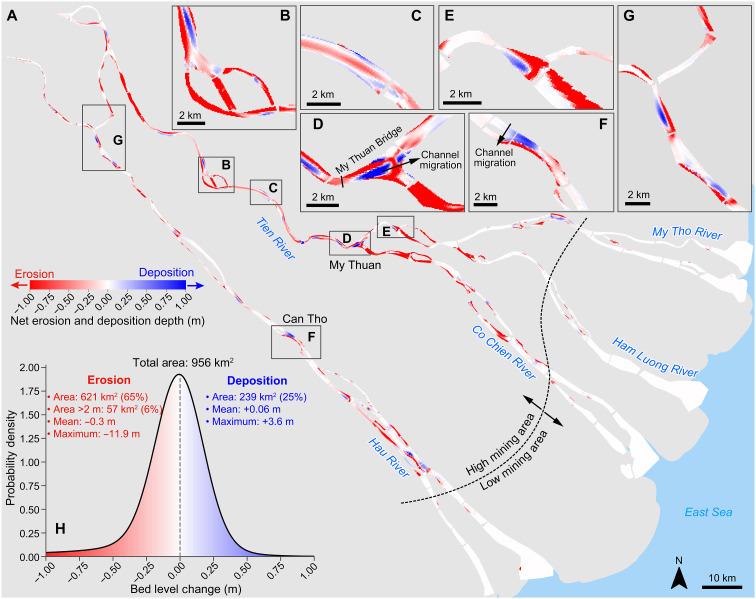
Net erosion and deposition induced by sand mining in the VMD between 2017 and 2021. (**A**) Spatial distribution of bed-level change (mining minus no-mining), showing widespread channel incision across the distributary network, particularly along the Tien and Hau rivers. Red indicates erosion and blue indicates deposition; the color scale is clipped at ±1 m to enhance visualization of spatial patterns. Insets (**B** to **G**) highlight representative reaches exhibiting localized incision, deposition, and channel migration associated with mining intensity. (**H**) Probability density distribution of bed-level change across the 956-km^2^ active channel area. Erosion dominates, affecting 621 km^2^ (65%) with a mean bed lowering of −0.30 m and maxima reaching −11.9 m; deep incision (>2 m) occurs over 57 km^2^ (6%). Deposition occurs over 239 km^2^, with a mean of +0.06 m and local maxima up to +3.6 m.

The sediment deficit produced extensive geomorphic alteration across the VMD channel network over the 2017 to 2021 simulation period. Bed-level change (Fig. 2, A to G), calculated as the difference between mining and no-mining simulations, isolates the effect of extraction and shows a strong spatial association (*CC* ≈ 0.80) between mining intensity, initial water depth, and geomorphic response. Approximately 65% of the active channel area experienced net incision, with a mean bed lowering of −0.30 m and a maximum scour depth of −11.9 m (Fig. 2H). Of this area, 6% (57 km^2^) exceeded −2-m incision, marking the most severely degraded reaches. In contrast, ~25% of the channel area showed limited deposition, with a mean aggradation of ~+0.06 m, while ~10% remained nearly unchanged (absolute change <0.5 mm), indicating system-wide but spatially uneven morphological adjustment (Fig. 2H). The most extensive incision was concentrated in the upper distributaries, with localized hot spots along the Tien and Hau rivers (Fig. 2, A to G).

Mining was predominantly located in initially shallow reaches, where extraction deepened the channel bed (fig. S7, C and D). Compared with the no-mining scenario, mining increased the area of the deepest channel class (bed elevation <−16 m) by 18.5%, while intermediate deep classes (−16.0 to −14.7 m and −14.7 to −13.3 m) expanded by 8.6 and 3.2%, respectively. In contrast, the shallowest class (>−4.7 m) declined by ~9% (fig. S7F). The My Thuan reach (Fig. 2D) shows that high-intensity mining in initially shallow reaches produced localized deep pits and widespread incision, whereas deeper zones with lower mining intensity exhibited smaller changes or weak deposition. The spatial coupling of erosion and deposition in this reach suggests local lateral channel adjustment, with intensified outer-bank erosion potentially promoting channel migration. Lower delta and estuarine reaches, under stronger tidal-marine influence, showed limited deposition or negligible change. Overall, the kernel-density distribution (Fig. 2H) is moderately skewed toward negative values, confirming incision dominance. This incision increased channel capacity, steepened banks, and enhanced lateral erosion along the main distributaries, thereby promoting tidal propagation and salinity intrusion (*23*). These patterns indicate that sand mining is a key driver of morphological change within the modeled framework.

#### Temporal dynamics and sediment balance

**Fig. 3.**
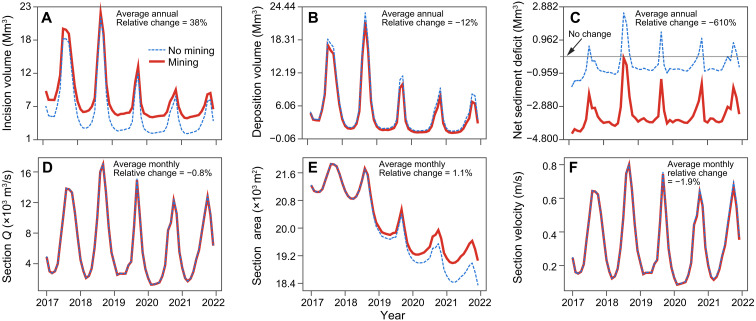
Geomorphic and hydraulic evolution from sand mining in the VMD. (**A**) Monthly incision volume (Mm^3^) from 2017 to 2021 under no-mining (blue) and sand mining (red) scenarios; average annual relative change shown in the panel. (**B**) Monthly deposition volume (Mm^3^) under the same scenarios, highlighting reduced deposition under sand mining. (**C**) Monthly net sediment deficit (Mm^3^), highlighting a persistent negative sediment balance. (**D**) Discharge (×10^3^ m^3^/s) at the Can Tho cross section under no-mining and sand mining conditions; average monthly relative change shown in the panel. (**E**) Wetted area (m^2^ × 10^3^) at the same cross section, showing an average monthly relative increase under sand mining (+1.1%). (**F**) Mean flow velocity (m/s), which decreases under sand mining (−1.9%) due to increased wetted area and reduced flow diversion from the Tien to the Hau River.

While spatial patterns captured the cumulative geomorphic response (Fig. 2), temporal analysis revealed how mining-driven sediment imbalance evolved through time. Direct scenario comparison of annual volumetric changes (Fig. 3, A to C) showed that sand mining fundamentally altered the temporal sediment balance of the system. Under no-mining conditions, incision and deposition exhibited strong seasonal variability and remained broadly balanced around monsoonal peaks. In contrast, the mining scenario disrupted this balance, producing persistently higher incision and reduced deposition throughout the simulation period. Total incision increased by an average annual relative change of ~38%, while deposition decreased by ~12%, indicating a sustained shift toward net sediment loss (Fig. 3, A and B). This imbalance produced a persistent negative sediment budget, with net deficits increasing by up to ~610% relative to no-mining conditions (Fig. 3C). Under no-mining conditions, the system remained closer to equilibrium, whereas the mining scenario produced a continuous deficit, reaching approximately −4.8 Mm^3^ in early 2017 and remaining consistently negative thereafter (Fig. 3C). These trends indicate that natural seasonal sediment replenishment was insufficient to offset extraction-induced losses. The temporal evolution explains the spatial patterns observed in Fig. 2: Widespread incision reflects the cumulative effect of sustained sediment deficits, while limited deposition corresponds to reduced sediment availability. These findings demonstrate that sand mining imposes a persistent and compounding sediment imbalance across the VMD.

### Hydrodynamic responses to channel incision

#### Flow redistribution and hydraulic capacity changes

Analysis of 14 cross sections (CS1 to CS14) along the VMD distributaries revealed that sand mining altered hydrodynamics, redistributing discharge, increasing wetted cross-sectional area, and reducing mean flow velocity (Fig. 3, D to F, for Can Tho; figs. S8 to S10 for all cross sections). The largest change occurred at the Vam Nao connection (CS3), where mean discharge decreased by ~53 m^3^/s (−1.18%, *P* < 0.001), indicating reduced transfer from the Tien to the Hau River system (fig. S8). Consistent reductions of ~53 m^3^/s were observed along the Hau River (Long Xuyen, Can Tho, and the river mouth, −0.83%), while discharge increased within the Tien River at Cao Lanh and My Thuan (+53 m^3^/s, +0.96%). Downstream, flow was preferentially redistributed into the Co Chien River (+85 m^3^/s, +3.9%), whereas the My Tho and Ham Luong distributaries showed decreases of ~25 m^3^/s (−1.07%) and 6.7 m^3^/s (−0.66%), respectively (fig. S8). This redistribution was accompanied by increases in wetted cross-sectional area. Mean expansions reached 860 m^2^ (+11.8%, *P* < 0.001) at the Ham Luong inlet (CS9), 450 m^2^ (+3.4%) at My Thuan (CS7), and 430 m^2^ (+3.3%) at Cao Lanh (CS5) (fig. S9). These geometric changes corresponded to reduced velocities, with the largest decline at the Ham Luong inlet (−0.018 m/s, −12%), and smaller but consistent decreases at Cao Lanh (−0.0098 m/s, −2.3%) and My Thuan (−0.0098 m/s, −2.2%) (fig. S10). The Co Chien River inlet branches were the main exception, showing velocity increases (+0.0047 m/s, +2.9%) where discharge gained outweighed area expansion. These increases in wetted cross-sectional area indicate enhanced local flow capacity, allowing channels to convey similar or redistributed discharge at lower velocities. These adjustments show that mining-driven changes in channel geometry control discharge redistribution and velocity response.

#### Tidal amplification and shifting energy partitioning

To isolate the high-frequency tidal response, we applied a Godin-filter decomposition to discharge time series at 14 cross sections (CS1 to CS14), revealing increased tidal variability in middle distributaries where incision was most pronounced (table S2). Tidal discharge variability (*TDV*) increased across middle distributaries, including Can Tho (+2.0%), My Thuan (+3.0%), Cao Lanh (+3.2%), and Co Chien In (+4.8%), with smaller increases at Long Xuyen (+1.5%), Ham Luong In (+1.3%), and My Tho In (+0.3%). In contrast, *TDV* changes at Vam Nao (−0.008%), downstream estuarine outlets (Hau Out, Co Chien Out, Ham Luong Out, and My Tho Out; ≤0.3%), and upstream boundaries (Tan Chau and Chau Doc; ≤ 0.02%) remained minimal, suggesting that tidal amplification was concentrated mainly in incised middle distributaries. Corresponding absolute increases in the tidal-dominance ratio, defined as the proportion of total-flow variance driven by tides, further indicate enhanced tidal influence, with increases of 0.007 to 0.009 in middle reaches including Can Tho, My Thuan, Cao Lanh, and Long Xuyen and 0.004 to 0.006 at Vam Nao and Ham Luong In. These changes occurred despite only small changes in mean discharge and were associated with reduced hydraulic damping following channel deepening and cross-sectional area expansion. This behavior reflects continuity-driven tidal amplification, whereby deeper channels transmit larger tidal fluxes inland (*38*). Consequently, channel incision not only redistributes discharge laterally but also reconfigures the river-tidal energy balance, increasing sensitivity to tidal propagation and salinity intrusion, as observed in other incision-affected systems (*39*).

### Sediment transport and composition response

#### Redistribution of sediment fluxes

**Fig. 4.**
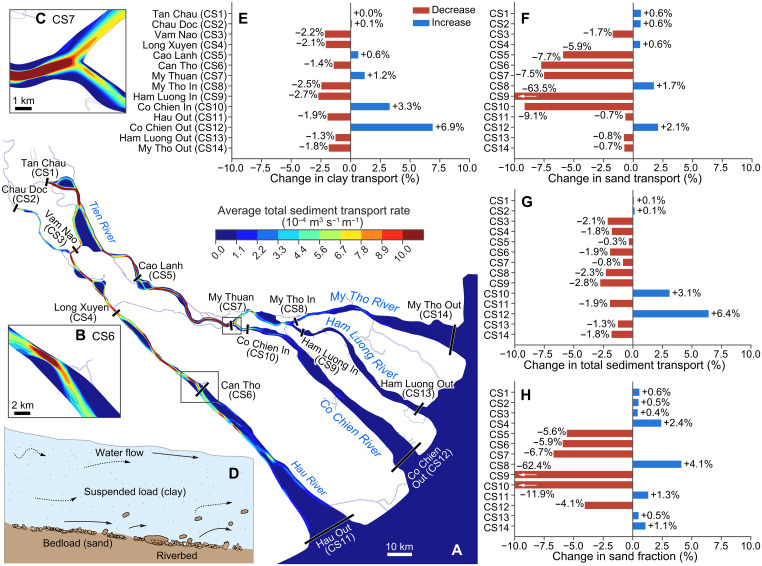
Effects of sand mining on sediment transport dynamics and load composition in the VMD. (**A** to **C**) Spatial distribution of time-averaged total sediment transport rate, showing concentration of flux in the upper Tien and Hau rivers and a downstream decline toward distributary mouths. (**D**) Conceptual schematic illustrating sediment transport pathways, with suspended load (clay-dominated) and bedload (sand-dominated) components. (**E** to **G**) Percentage change in cumulative cross-sectional fluxes of clay, sand, and total sediment across 14 cross sections (CS1 to CS14) under mining relative to no-mining conditions; bars indicate increases (blue) and decreases (red). (**H**) Change in sand fraction within the total transported load, showing spatial variations in sediment composition across the distributary network.

Spatial patterns of sediment transport magnitude provide the baseline context for these changes (Fig. 4, A to C). Transport is concentrated along the main Tien and Hau rivers, where fluxes are highest, and decreases toward downstream distributaries. Lower transport rates dominated the distributary branches and near-outlet sections, indicating reduced sediment conveyance capacity. This spatial structure is consistent with the dominant transport mechanisms illustrated in Fig. 4D, where sand is primarily transported as bedload and clay as suspended load. Following the discharge redistribution at the Vam Nao connection, sediment fluxes showed a similarly structured response across the VMD channel network (Fig. 4, E to H). At the upstream boundaries (Tan Chau and Chau Doc), changes remained negligible (within ±1%), indicating limited upstream response to downstream mining. At Vam Nao, clay and total sediment transport decreased (−2.2 and −2.1%), while sand transport declined only slightly (−1.7%), leading to a marginal increase in sand fraction. The reduction in sand transport is consistent with spatial redistribution of bed shear stress following mining-induced incision, with shear-stress decreases dominating during the dry period and localized increases occurring near channel-adjustment zones (fig. S11). Along the Hau branch downstream of Vam Nao, reductions propagated downstream, with consistent decreases in clay (−1.4 to −2.1%) and total sediment (−1.8 to −1.9%), while sand transport shows stronger declines (−5.9 to −9.1%). In contrast, along the Tien branch, clay transport increased slightly (0.6 to 1.2%), whereas sand transport decreased markedly (−5.9 to −7.5%), resulting in weak net reductions in total sediment transport. Downstream of My Thuan, the three distributaries responded differently (Fig. 4, E to H). The Ham Luong branch shows the strongest reduction, with sand transport decreasing by 63.5% and sand fraction by 62.4% at the inlet. The My Tho branch showed moderate declines in clay and total sediment but slight increases in sand transport and sand fraction at the inlet. In contrast, the Co Chien branch showed increases in clay (3.3 to 6.9%) and total sediment transport (3.1 to 6.4%), despite reductions in sand transport at the inlet (Fig. 4, E and F). Overall, reductions were strongest for sand transport, whereas clay transport showed smaller and more variable changes, including downstream increases. These contrasting responses produce spatially variable shifts in sediment composition across the distributary network.

#### Bed-material composition response

Building on the sediment transport response, the simulated bed composition showed a spatially heterogeneous adjustment to sand mining (fig. S12). Sand-fraction changes were nearly evenly distributed, with 51% of the channel area showing depletion and 49% showing enrichment, while less than 0.02% remained unchanged. The magnitude of change was substantial, with pronounced local variability. Depleted areas showed a mean reduction of −0.77 percentage points (pp), whereas enriched zones showed a mean increase of 0.91 pp (fig. S12). Sand depletion exceeding 0.5 pp affected 21.3% of the channel area (181 km^2^), with local extremes reaching −38.3 and 35.4 pp (fig. S12). Spatially, increases in sand fraction occurred mainly in deepened reaches and channel junctions, consistent with exposure of coarser substrate or selective removal of finer material. In contrast, adjacent areas showed reduced sand fraction, likely due to fine-sediment deposition in lower-energy zones. At the delta scale, these opposing responses produced a modest net fining, with mean sand fraction decreasing from ~50.8 to 49.6% (−1.2 pp). The kernel-density distribution was unimodal and centered near zero but slightly shifted toward negative values, indicating spatially heterogeneous but slightly fining-dominated changes in bed composition (fig. S12). These patterns are consistent with sand mining–induced bed disturbance and sediment sorting, where extraction, selective transport, and local flow divergence can modify bed grain-size composition and produce localized coarsening or fining in sand-bed rivers (*40*).

### Amplification and inland extension of salinity intrusion

**Fig. 5.**
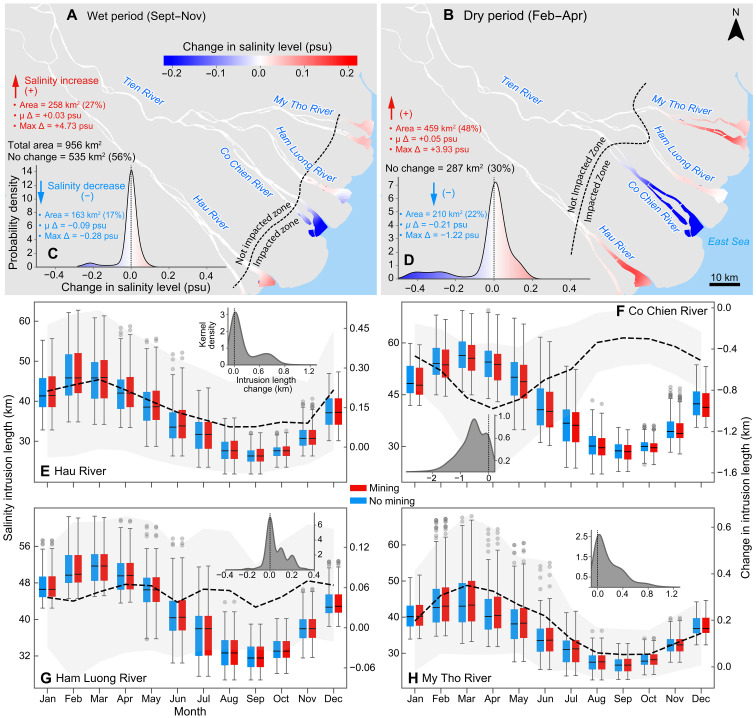
Seasonal and spatial amplification of salinity intrusion under sand mining in the VMD. Spatial distributions of salinity change (mining–no-mining) averaged over the (**A**) wet period (September to November) and (**B**) dry period (February to April). Blue indicates salinity decrease and red indicates increase; the color scale is clipped at ±0.2 psu for visualization, and the full range is shown in the map annotations. Kernel-density insets (**C** and **D**) show the frequency distribution of salinity change. During the dry season, salinity amplification extends ~50 to 60 km inland along the Hau, Ham Luong, and My Tho rivers, with local maxima up to 3.9 psu, whereas wet-season changes remain weak (<0.05 psu) and confined to estuarine reaches (~30 to 40 km). (**E** to **H**) Monthly salinity-intrusion length for the Hau, Co Chien, Ham Luong, and My Tho rivers from 2017 to 2021. Blue and red boxplots denote no-mining and mining scenarios, respectively. Mining increases intrusion lengths in the Hau (+0.20 km), Ham Luong (+0.10 km), and My Tho (+0.30 km) but shortens intrusion length in the Co Chien (−0.50 km) because of freshwater redistribution.

Salinity simulations, expressed as differences between mining and no-mining scenarios, revealed that sand mining amplified and extended saltwater intrusion across much of the VMD, increasing both seasonal intensity and spatial extent (Fig. 5, A to H). The effect was most pronounced during the dry season (February to April), when river discharge is low and tidal forcing dominates. During this period, salinity increases were more spatially extensive, affecting 459 km^2^ (48%) of the domain, with a mean increase of 0.05 practical salinity units (psu), mainly along the Hau, Ham Luong, and My Tho distributaries. Changes extended up to ~70 km inland along these distributaries, with local maxima reaching ~3.9 psu (Fig. 5B). In contrast, during the wet season (September to November), mean salinity changes remained small, with 56% of the domain showing negligible change and salinity-increase areas averaging only 0.03 psu, largely confined to lower estuarine and coastal reaches (Fig. 5A). The Co Chien River showed a localized salinity decrease, particularly during the dry season, likely reflecting redistribution of freshwater discharge toward this branch. Overall, ~30% of the dry-season domain showed negligible change concentrated in upstream freshwater zones, while the remaining ~22%, primarily around the Co Chien River, exhibited localized salinity decreases with a mean decrease of −0.21 psu (Fig. 5B).

Kernel-density distributions (Fig. 5, C and D) show a clear right-skewed pattern during the dry season, indicating a delta-wide shift toward higher salinity under mining, whereas distributions during the wet season remain narrowly centered around zero, reflecting minimal change. Changes in salinity intrusion length, defined using the 2-psu threshold, further confirm this amplification. Time-series analysis from 2017 to 2021 shows mean landward extension in three distributaries: Hau (0.20 km), Ham Luong (0.10 km), and My Tho (0.30 km) (Fig. 5, E to H). Maximum instantaneous excursions reached ~0.8 to 1.5 km, and intrusion-length changes were statistically significant (*P* < 0.05). Under mining conditions, intrusion-length distributions shifted toward greater inland penetration, rather than only increasing the mean, indicating more frequent landward excursions during dry-season low-flow conditions. Kernel-density estimates further reveal positively skewed distributions, reflecting episodic but pronounced landward excursions, particularly in the Hau and My Tho rivers. In contrast, the Co Chien River showed a localized reduction in intrusion length (−0.50 km), consistent with freshwater redistribution toward this branch. Overall, these patterns indicate that mining-induced changes in channel geometry and flow partitioning amplified salinity intrusion in most distributaries, while producing localized salinity relief in the Co Chien system. Temporal patterns suggest incomplete seasonal recovery in some distributaries, with mining-related intrusion-length increases persisting beyond the peak dry season. This indicates that continued channel deepening may increase the delta’s sensitivity to future salinity intrusion (see Discussion).

## DISCUSSION

### Interconnection of mining-driven processes

**Fig. 6.**
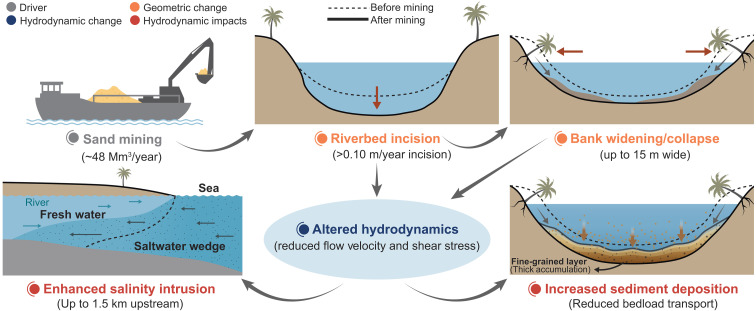
Conceptual diagram of the systemic impacts of sand mining in the VMD based on counterfactual modeling that isolates the effects of sand extraction from other drivers. The results reveal a cascading degradation of the river system: Intensive sand mining (~48 Mm^3^/year) directly drives mean riverbed incision (~0.10 m/year) and subsequent bank widening (up to 15 m wide). These morphological changes alter hydrodynamics by reducing flow velocity and shear stress, thereby decreasing sediment transport capacity, promoting deposition, and amplifying tidal penetration, ultimately enhancing salinity intrusion (up to ~1.5 km further inland). These interconnected feedback loops threaten geomorphic stability and freshwater security.

Our counterfactual simulations demonstrate that sand mining acts as a key driver of system-wide reorganization in the VMD, linking morphodynamic, hydrodynamic, sediment transport, and salinity processes through mechanistically coupled and mutually reinforcing feedbacks (Fig. 6), consistent with growing evidence that large rivers are increasingly reshaped by human pressures (*7*, *9*). Rather than operating as isolated responses, these processes emerge as an integrated system adjustment to sustained sediment extraction (*41*). The system response is initiated by intensive sand removal (~48 Mm^3^/year), which generates a persistent sediment deficit exceeding natural supply by a factor of 6 to 15, comparable to sediment starvation reported across the Mekong system due to dam trapping and extraction (*8*, *20*). This imbalance drives widespread channel incision, affecting ~65% of the active channel area, with a cumulative mean bed lowering of ~−0.30 m over the 2017 to 2021 period (equivalent to ~0.10 m/year on average), consistent with observed rapid bed lowering in the Mekong Delta (*17*, *18*). The resulting modification of channel geometry directly controls hydrodynamic response. Increases in cross-sectional area up to 11.8% and associated reductions in flow velocity (up to −12%) indicate a transition toward a hydraulically adjusted system capable of conveying similar discharge under lower energy conditions. This adjustment also redistributes discharge across the distributary network, with reduced transfer from the Tien to the Hau system (~−53 m^3^/s) and enhanced routing toward alternative branches such as the Co Chien. These changes demonstrate that incision modifies not only local hydraulics but also basin-scale flow partitioning.

Hydrodynamic reorganization subsequently drives a fundamental shift in sediment transport dynamics. Reduced flow velocity and bed shear stress limit the capacity for bedload transport, producing a systematic decline in sand flux (typically −6 to −10%) (*27*, *31*), with larger localized losses at Ham Luong In, while clay transport exhibits smaller and more spatially variable changes. This selective reduction leads to a decoupling of coarse and fine sediment transport, as reported in sand mining–affected river systems, with the system transitioning toward a suspension-dominated regime (*23*). At the bed scale, this manifests as spatially heterogeneous but net fining, with mean sand fraction decreasing from ~50.8 to 49.6%. The trapping of sand within deepened channels and mining pits further reinforces incision by limiting downstream sediment replenishment, establishing a self-reinforcing feedback between morphology, hydraulics, and sediment transport. Concurrently, mining-induced channel deepening alters the balance between fluvial and tidal forcing. Tidal decomposition reveals increased tidal variability (2 to 4% across mid-delta distributaries) and enhanced tidal dominance, reflecting reduced hydraulic damping and more efficient inland propagation of tidal energy.

This shift in energy partitioning provides a direct pathway for saltwater intrusion, resulting in statistically significant amplification and inland extension of salinity (*13*). Dry-season intrusion increases across most major distributaries, with mean landward extensions of 0.10 to 0.30 km and maximum excursions reaching ~0.8 to 1.5 km, while Co Chien shows localized salinity relief due to freshwater redistribution. These processes are not sequential but dynamically coupled. Channel incision modifies hydraulics, which in turn regulates sediment transport and tidal propagation, while changes in sediment composition and salinity feedback onto channel stability and flow dynamics. The system response therefore represents an emergent property of interacting processes rather than the sum of individual effects. This explains why localized extraction produces basin-scale impacts and why single-factor interpretations underestimate system sensitivity. Within this integrated framework, sand mining can be directly linked to the observed reorganization of delta dynamics. By isolating mining effects through counterfactual simulations, we demonstrate that incision-driven geometric changes act as the central control, propagating through hydrodynamic adjustment, sediment redistribution, and salinity intrusion. This mechanistic linkage highlights that effective management cannot target individual processes in isolation but must account for the interconnected nature of sediment budgets, channel morphology, flow dynamics, and freshwater availability.

Thus, channel incision not only redistributes discharge laterally but also intensifies salinity intrusion and geomorphic instability. In the VMD, this human-induced reconfiguration heightens salinity-intrusion risk along the Hau River and increases flood vulnerability in the Co Chien system, posing critical challenges to long-term stability and resilience (*17*). Building on this, our integrated framework provides two critical capabilities. First, it enables clear attribution: By comparing mining scenarios with a counterfactual no-mining baseline, we isolate the mining signal and demonstrate that the observed changes are mechanistically consistent and direct outcomes of extraction-induced incision. Second, it enables system-level inference: Analyzing any component in isolation underestimates the systemic response, as the net effect emerges from interacting processes. For instance, the magnitude of bed fining cannot be predicted from extraction volumes alone but depends on mining-induced reductions in velocity that limit downstream sand transfer. Similarly, salinity intrusion reflects not only oceanic forcing but also internally modified and continuously evolving bathymetry. This integrated perspective explains why management strategies targeting a single metric (e.g., extraction volume alone) often fail. Instead, effective mitigation must account for cross-process feedbacks, addressing the cascade from sediment deficit and incision to altered hydrodynamics, sediment sorting, and salinization to ensure long-term resilience of the VMD.

### Quantitative attribution of sand mining relative to other drivers

The counterfactual modeling framework enables direct attribution of system change to sand mining by isolating extraction as the only varying factor between scenarios. Under identical boundary conditions, all differences in morphology, hydrodynamics, sediment transport, and salinity therefore represent intrinsic responses to mining-induced channel modification. This provides a first-order quantification of the standalone contribution of sand mining within a multidriver system. At the basin scale, model results indicate a mean bed lowering of ~0.10 m/year attributable to sand mining under present-day conditions. When compared with basin-scale bathymetric observations of ~0.30 m/year, this suggests that sand mining contributes ~25 to 30% of total riverbed incision. In addition, land subsidence, reported at rates of ~1 to 5 cm/year in parts of the delta (*42*), further contributes to relative bed lowering and enhances the apparent magnitude of observed incision, although it operates through different processes than direct sediment removal. While incision is primarily governed by basin-scale sediment reduction (*3*, *4*), sand mining represents a substantial and independently quantifiable component of channel deepening. This interpretation is consistent with observational studies that attribute widespread incision in the VMD to the combined effects of upstream sediment trapping and in-channel extraction (*21*).

Salinity intrusion provides a complementary metric for relative attribution. In the delta, it is controlled by the combined influence of sea level rise, altered river discharge, land subsidence, and human interventions such as sand mining, which together regulate the landward penetration of saline water (*13*, *41*, *43*, *44*). Reported subsidence rates of up to ~1 to 5 cm year^−1^ in parts of the delta further amplify relative sea level rise, accelerating long-term salinity intrusion (*43*). In contrast, river discharge exerts the primary short-term control, with reduced dry-season flows weakening the freshwater gradient and allowing deeper inland salinity intrusion, while human interventions such as upstream dam operations further modify both the magnitude and timing of discharge, prolonging low-flow conditions and enhancing intrusion (*45*). Model results indicate that sand mining alone increases mean salinity by ~0.06 to 0.25 psu over a 5-year period, corresponding to an annualized increase of ~0.01 to 0.06 psu/year. When compared with reported system-wide salinity trends of ~0.2 to 0.5 psu in the VMD (*46*), this implies that sand mining contributes ~16 to 30% of the total observed rate of salinity increase under present-day conditions. Notably, this relative contribution is consistent with the magnitude of mining-induced riverbed incision (~25 to 30%), indicating that channel deepening associated with extraction translates into comparable increases in salinity intrusion. The remaining ~70 to 85% reflects the influence of other drivers, primarily upstream sediment trapping and reductions in freshwater discharge, together with contributions from subsidence and sea level rise.

### Implications for freshwater security and delta stability

Salinity responses to sand mining exhibit pronounced spatial heterogeneity across the distributary network, reflecting mining-induced reorganization of flow pathways rather than uniform system-wide change. While most distributaries, including the Hau, Ham Luong, and My Tho rivers, experience enhanced salinity intrusion (*33*, *45*–*48*), the Co Chien River shows a localized reduction. This apparent improvement does not indicate recovery but instead reflects discharge redistribution, whereby channel deepening preferentially routes fresh water toward the Co Chien branch, reducing salinity locally while intensifying intrusion elsewhere. This pattern highlights a fundamental system-level trade-off, where localized freshwater gains occur at the expense of broader freshwater loss.

These dynamics limit the effectiveness of upstream discharge regulation as a standalone mitigation strategy. Although increased freshwater release can reduce salinity intrusion, its efficiency is constrained by channel geometry and flow partitioning, requiring progressively larger discharge volumes to offset equivalent salinity increases. Moreover, additional flow is unevenly distributed across distributaries, preventing uniform mitigation. This behavior is consistent with hydrodynamic theory linking salinity intrusion to the balance between freshwater discharge and channel depth (*49*). Within this framework, sand mining emerges as a substantial and previously underquantified driver of salinity intrusion, contributing ~16 to 30% of the total increase under present-day conditions. This contribution is critical for freshwater security, as even modest salinity increases can disproportionately reduce water availability, agricultural productivity, and ecosystem functioning, particularly during dry-season low-flow conditions (*14*). Sustained sand mining also threatens long-term delta stability. The VMD’s low elevation (~0.8 m) and ongoing relative sea level rise (~3 to 5 mm/year) and subsidence (~1 to 5 cm/year) make it highly sensitive to elevation loss. Under these conditions, mining-induced incision and sediment deficits (6 to 15 times supply) reduce sediment storage and limit geomorphic recovery, reinforcing channel deepening and salinity intrusion. These combined effects further weaken freshwater resilience, increasing vulnerability to salinization and long-term risks to water resources, agriculture, and habitability (*13*, *50*).

### Limitations and future research directions

While the integrated Delft3D-FM framework demonstrates robust skill in reproducing hydrodynamic, sediment, and salinity dynamics, several limitations inherent to large-scale morphodynamic modeling remain. First, the use of a depth-averaged (2D) framework limits representation of vertical processes such as stratification, estuarine circulation, and near-bed sediment dynamics, which may influence fine-sediment transport and salinity intrusion under low-flow conditions. Second, the upstream model boundary is located below the tidal limit near Phnom Penh, preventing full resolution of tidal damping and current reversals in upstream reaches and introducing uncertainty in sediment transport pathways within the fluvial-tidal transition zone. Third, sediment is represented using a simplified two-fraction scheme (cohesive and noncohesive), which limits representation of grain-size variability, selective transport, and sediment sorting processes. In addition, the bed is treated as a vertically mixed layer, which does not resolve stratification or delayed sediment availability. Consequently, simulated changes in bed-material composition may not fully capture subsurface heterogeneity, particularly in areas of intense extraction. Fourth, bank erosion is represented using empirical relationships rather than a fully coupled geotechnical model, limiting representation of feedbacks between basal scour, pore-pressure dynamics, and bank failure. Fifth, model accuracy depends on data availability, with uncertainties arising from limited sediment observations and the difficulty of capturing localized or unreported sand mining activities (*51*).

While the counterfactual framework isolates sand mining impacts under present-day conditions, it does not provide a full quantitative ranking of interacting drivers such as dam regulation, sea level rise, land subsidence, and climate variability. Resolving their relative contributions would require a dedicated factorial modeling framework in which drivers are varied independently and in combination, which is particularly important for management applications. In addition, the representation of sand extraction as spatially distributed and temporally continuous smooths short-term variability and does not resolve localized processes such as knickpoint formation and upstream migration. Incorporating temporally variable extraction scenarios would improve representation of these dynamics. More broadly, future work should focus on improving process representation and decision-support capability through targeted three-dimensional simulations, improved sediment characterization, enhanced bank erosion modeling, and expanded observational datasets. Despite these limitations, the framework provides a strong foundation for next-generation decision-support systems. By integrating scenario-based simulations with process-based thresholds, future applications can identify sustainable extraction limits, delineate hydraulically sensitive “no-go” zones, and support early-warning systems for salinity intrusion. The transferability of this approach to other sediment-limited deltas further highlights its potential for global-scale assessment and management of sand mining impacts (*52*).

## MATERIALS AND METHODS

### Study area and modeling domain

The VMD is a low-lying fluvial-deltaic system in southern Vietnam, covering ~40,000 km^2^, with most elevations below 2 m above mean sea level (*41*). The Mekong River bifurcates into the Tien and Hau distributaries after entering Vietnam, which form a dense and interconnected channel network that discharges into the East Sea through multiple estuarine outlets (Fig. 1). The hydrodynamic regime is controlled by the interaction of seasonal river discharge, semidiurnal tides (2- to 4-m range), and dry-season salinity intrusion extending up to 60 to 70 km inland during the dry season. These coupled fluvial-tidal processes strongly influence sediment transport, channel morphology, and estuarine circulation across the delta. To capture these system-scale dynamics, the modeling domain spans the full fluvial-estuarine-marine continuum, extending from the upstream boundaries at Tan Chau and Chau Doc to the ocean (figs. S13 and S14). The domain includes all major distributaries, estuarine outlets, and adjacent coastal waters, enabling simulation of tidal propagation, salinity intrusion, and sediment redistribution. An unstructured flexible mesh was used to represent the complex channel-coastal geometry, with grid resolution refined to ~10 m in channels, bifurcations, and sand mining hot spots, and coarsened to ~8 km offshore (figs. S13 and S14). Monitoring stations and cross sections (CS01 to CS14) were incorporated for calibration, validation, and analysis of flow and sediment partitioning (fig. S13). Grid quality satisfies Delft3D-FM criteria (orthogonality <0.020; smoothness <3), ensuring numerical stability (fig. S14).

### Sand mining representation and spatial delineation

Sand mining was represented using 131 spatially explicit extraction zones distributed along the main channels of the VMD (fig. S15), based on reported sand mining activity hot spots identified in (*11*). The zoning scheme was designed to capture spatial heterogeneity while maintaining computational efficiency, with smaller zones in areas of intense extraction (e.g., upstream of Can Tho and My Thuan) and larger zones in regions of diffuse activity. Zone size was further refined in geomorphically complex areas, such as channel confluences, to better resolve local impacts. The delineated zones cover ~960 km^2^ of channel bed, with individual areas ranging from 0.29 to 77 km^2^. Each zone was assigned an extraction rate based on reported annual removal volumes ranging from negligible to 3.3 Mm^3^/year, totaling ~48 Mm^3^/year across the delta (fig. S15). For reach-scale analysis, the 131 zones were aggregated into six major river reaches (R1 to R6), providing a multiscale framework for evaluating the influence of spatially variable extraction on morphology, sediment budgets, and hydrodynamics.

### Numerical model integration and development

This study used Delft3D Flexible Mesh (version 2025.01), integrating hydrodynamic, salinity, sediment transport, and morphodynamic modules to simulate coupled river-estuary-coastal processes in the VMD. This model setup resolves interactions between upstream discharge, tidal forcing, and sediment transport at each time step (*53*). The hydrodynamic model was configured using observed hourly discharge at Tan Chau and Chau Doc, with marine boundary conditions derived from TPXO 8.0 tidal constituents [(*54*); Supplementary Text 2]. The salinity model was coupled to hydrodynamics, with upstream freshwater (0 psu) and marine boundary conditions set to 33 psu. A depth-averaged (2D) configuration was adopted to simulate horizontal advection and estuarine circulation (Supplementary Text 3). Sediment transport was represented using a two-class system of cohesive (clay/silt) and noncohesive (sand) fractions. Upstream sediment input was prescribed as a fixed concentration (95% cohesive, 5% noncohesive), scaled with discharge. Cohesive transport included salinity-dependent settling, while sand transport accounted for both bedload and suspended components, with bedload simulated dynamically using an equilibrium approach (Supplementary Text 4). Bathymetry was derived from 2017 river surveys and supplemented offshore with GEBCO 2019 data (*55*). Morphodynamic feedback during simulations adjusted initial discrepancies, ensuring consistency over the model period (Supplementary Text 5). A two-stage initialization was applied, including a spin-up phase to stabilize hydrodynamics, salinity, and sediment conditions. The bed was initialized as a two-layer system (sand overlain by clay), which evolved into a mixed active layer through sediment exchange processes. Outputs from this phase were used as initial conditions for the main simulations (fig. S16 and Supplementary Text 6).

### Model calibration and validation

Model calibration and validation were performed sequentially for hydrodynamics, salinity, and sediment transport modules. Hydrodynamic calibration focused on bed roughness (Manning’s ***n*** = 0.017 to 0.019, spatially varied) and turbulence parameters to reproduce observed water levels and discharge at key monitoring stations. Salinity calibration targeted dry-season intrusion patterns, with horizontal eddy viscosity and turbulence parameters adjusted to reproduce observed salinity gradients. Sediment transport was calibrated against observed suspended sediment concentrations from monitoring stations and Mekong River Commission datasets (*56*). Cohesive sediment parameters, including critical shear stress and settling velocity, were adjusted on the basis of field measurements. Noncohesive (sand) transport was simulated using the Van Rijn formulation (*57*). Morphological validation was conducted by comparing simulated bathymetric changes from 2017 to 2021 with independent field measurements collected in 2020, evaluating the model’s ability to reproduce channel evolution and bed-level change across both sand mining hot spots and the wider delta. For bathymetric validation, comparisons were restricted to overlapping wet-channel areas represented in both the survey data and the model grid. Cells affected by unresolved narrow water passages, dry-bank artifacts, or nonoverlapping survey coverage were manually inspected and excluded where necessary to ensure a like-for-like comparison between observed and simulated bathymetry. Model performance was assessed using standard statistical metrics, including WSS, Nash-Sutcliffe efficiency, *R*^2^, root mean square error, and percent bias. These metrics were computed at hourly, daily, and monthly scales across hydrological and sediment stations (table S3).

### Model simulation and hydrodynamic tidal decomposition analysis

To isolate the impacts of sand mining from other environmental drivers, we performed a controlled counterfactual scenario analysis using a calibrated Delft3D-FM model. We ran a mining scenario (2017 to 2021) representing contemporary conditions with observed sand extraction, and a counterfactual no-mining scenario in which all spatially explicit extraction inputs were removed while boundary conditions, including discharge, sediment load, tidal forcing, and sea level, were kept identical. We quantified changes in river-tidal dynamics by decomposing discharge time series at 14 cross sections (CS1 to CS14) into river and tidal components using a Godin-type three-pass 24-hour low-pass filter (Godin, 1972). From the filtered signals, we derived mean river discharge (*Q*_*river*_), tidal discharge (*Q*_*tidal*_), TDV, tidal variance (*E*_tide_), and tidal dominance ratio (*D*_tide_), defined as the proportion of tidal to total discharge variance. Detailed formulations and definitions of these metrics are provided in Supplementary Text 7 and table S4.

### Sand mining impact assessment

We quantified sand mining impacts by comparing mining and no-mining scenarios across four domains: (i) sediment budget and geomorphology, including bed-level change, sediment storage, and sediment deficits; (ii) hydrodynamics, including discharge redistribution, flow velocity, and cross-sectional area; (iii) sediment transport and composition, including sand and clay fluxes and bed-material composition; and (iv) salinity intrusion, including the extent and intensity of saltwater propagation. We further compared tidal-fluvial decomposition metrics between scenarios, including *E*_tide_, *Q*_*river*_, TDV, and *D*_tide_, to characterize changes in flow variability and river-tidal interactions. We also used the counterfactual differences to estimate the first-order relative contribution of sand mining to observed riverbed incision and salinity increase, with details presented in Discussion. Spatial impact areas were calculated by classifying grid cells according to positive, negative, or negligible mining-induced changes and summing the corresponding active-channel or deltaic-domain areas. Bed-material composition changes were quantified from the simulated active bed layer as differences in sand fraction between mining and no-mining scenarios. Salinity intrusion length was quantified along each distributary using the 2-psu isohaline threshold, and mining impacts were calculated as differences in intrusion length between scenarios.

## Supplementary Materials

### This PDF file includes:

Supplementary Text Figs. S1 to S16 Tables S1 to S4 References
