# Supplementary material for: Counterfactual modeling isolates sand mining impacts, revealing it as a key driver of Mekong Delta destabilization
Source: Sci Adv. 2026 Jul 8;12(28):eaef0558. doi: 10.1126/sciadv.aef0558 (PMC13344278; doi:10.1126/sciadv.aef0558)
Supplement: Supplementary file 1 — Supplementary Text Figs. S1 to S16 Tables S1 to S4 References [file sciadv.aef0558_sm.pdf]

Science Advances
AAAS

Supplementary Materials for

Counterfactual modeling isolates sand mining impacts, revealing it as a key driver of Mekong Delta destabilization

Sonu Kumar *et al.*

Corresponding author: Edward Park, edward.park@nie.edu.sg

Sci. Adv. **12**, eaef0558 (2026)
DOI: 10.1126/sciadv.aef0558

This PDF file includes:

Supplementary Text
Figs. S1 to S16
Tables S1 to S4
References

Supplementary Text

Supplementary Text 1: Integrated Model Performance

This section provides detailed validation results supporting the model-performance summary reported in the main text. Performance was evaluated for hydrodynamics, salinity, suspended sediment concentration, and bathymetry using standard skill metrics across hourly, daily, and monthly scales where applicable.

The hydrodynamic model exhibited strong skill in simulating both discharge and water levels across the VMD (fig. S1A, S1B, and S2 to S3). This performance indicates that the model adequately represents the main flow regime, providing a suitable foundation for simulating sediment transport and morphological changes associated with sand mining. Simulated and observed water levels agreed closely at hourly scales, with pooled all-station statistics of Willmott Skill Score (WSS) = 0.94, NSE = 0.78, R^2 = 0.80, and RMSE = 0.35 m. PBIAS was generally within ±15%, suggesting limited overall bias. At Can Tho, the model captured both tidal oscillations and seasonal flood peaks (NSE = 0.79, RMSE = 0.29 m), while seaward stations such as Dai Ngai and Binh Dai maintained WSS above 0.85, reflecting good performance in tide-dominated zones (fig. S2). Simulated discharges also tracked observed variability well, including diurnal tides and compound flood-tide interactions (fig. S1B and S3). Performance was high at both Can Tho and My Thuan, where hourly WSS exceeded 0.97, with NSE = 0.87 and PBIAS near -0.8% (fig. S1B). Minor discrepancies occurred during peak floods, likely due to unrepresented wind setup and residual uncertainties in downstream tidal forcing. Overall, hydrodynamic performance met or exceeded commonly used thresholds for large-river delta models, indicating that the calibrated hydrodynamics provide a reliable basis for subsequent salinity, sediment, and morphodynamic simulations (*33*–*35*).

The coupled hydrodynamic-salinity model reproduced the timing, magnitude, and seasonal variability of salt intrusion across the estuaries of the VMD (fig. S2C and S4). At An Thuan and other downstream stations, the model captured the progressive landward intrusion during the dry season and flushing during monsoon months. Upstream stations were not evaluated, as salinity is negligible under observed conditions. Model performance improved with temporal aggregation. At the daily scale, WSS = 0.91, NSE = 0.65, R^2 = 0.68, RMSE = 3.29 psu, and PBIAS = -6.5% (fig. S2C). Monthly aggregation further improved agreement WSS = 0.93, NSE = 0.71, R^2 = 0.75, RMSE = 2.84 psu, PBIAS = -5.7%, indicating that basin-scale controls on salinity dynamics were well represented. Hourly simulations were more variable due to tidal oscillations, sub-daily mixing, and wind forcing, while observed salinity measurements are typically surface-only, leading to discrepancies when compared with depth-averaged model outputs under stratified or neap-tide conditions. Daily and monthly validations therefore demonstrate good skill for seasonal salinity dynamics. Compared to earlier delta-wide studies (*33*, *58*), the model achieved similar or higher accuracy for long-term salinity simulation. This provides a suitable basis for evaluating how mining-induced morphological adjustments influence saltwater penetration into freshwater zones (*59*).

The sediment transport module was validated for suspended sediment concentration (SSC) dynamics at two key stations in the central delta: Can Tho on the Hau River and My Thuan on the Tien River, where continuous observational data were available (fig. S1D and S5). Although validation was limited to these two locations, they represent the two major distributaries of the VMD and are therefore useful indicators of system-scale suspended sediment behaviour. At Can Tho, the model captured seasonal flood peaks and dry-season minima with daily WSS = 0.81, NSE = 0.41, R^2 = 0.56, RMSE = 0.02 g/l, and PBIAS = 22.7% (fig. S5). At My Thuan, model performance was lower (WSS = 0.66, NSE = 0.17, R^2 = 0.57, RMSE = 0.04 g/l, PBIAS = -39.4%), likely reflecting greater hydrodynamic complexity from channel bifurcations and tidal interference, but remained within acceptable ranges for delta-scale sediment simulations (fig. S5). The combined daily results across both stations showed moderate skill (WSS = 0.71, R^2 = 0.57), with daily assessments outperforming hourly comparisons because tidal-stage sampling in observations limits direct comparability at higher frequencies (fig. S1D). Model PBIAS remained moderate (-32% overall), with underestimation most evident during high-concentration events when near-bed resuspension and vertical gradients are likely strongest. Although the 2D depth-averaged framework cannot fully resolve vertical gradients or tidal pumping effects, the model reproduced seasonal SSC cycles and captured broad event-scale variability, while peak concentrations remained more uncertain. This level of skill is comparable to other large-scale sediment modelling studies (*24*, *60*), supporting the model's use for assessing relative changes in sediment fluxes and budgets under mining and no-mining scenarios.

Morphodynamic validation provided an additional spatial check on model performance. Agreement in bathymetric patterns provides a spatially distributed assessment of the plausibility of simulated sediment redistribution, beyond point-based SSC validation. The morphodynamic module reproduced broad channel morphology and key reach-scale patterns in observed bathymetry (fig. S1E and F, and S6). Along representative transect ST24 near My Thuan, where intensive sand mining occurs, the simulated and observed profiles in 2020 were closely aligned (WSS = 0.94, NSE = 0.74, RMSE = 2.53 m)(fig. S1F). At the delta scale, validation against 3,830 cross-sectional points across 165 transects showed domain-scale agreement of WSS = 0.81, NSE = 0.48, R^2 = 0.49, RMSE = 4.06 m, and PBIAS = 3.2%. Based on absolute depth differences between simulated and observed bathymetry, 38.3% of validation points differed by ≤ 1 m, 20.3% differed by 1−2 m, 28.5% differed by 2−6 m, and 12.9% differed by > 6 m. Cumulatively, 58.6% of points

were within ±2 m and 87.1% were within ±6 m. Larger mismatches were mainly associated with narrow, poorly surveyed, or geometrically complex channels where bathymetric interpolation and numerical smoothing were required (fig. S6). These localized mismatches indicate remaining uncertainty in small-scale bathymetric representation but did not dominate the domain-scale performance. Overall, the validation indicates that the model is suitable for evaluating system-scale and reach-scale relative differences between mining and no-mining scenarios, while local-scale bathymetric predictions should be interpreted with appropriate caution.

Supplementary Text 2: Hydrodynamic Boundary Conditions

Hourly discharge data from the Mekong River Commission (MRC) were prescribed at Tan Chau and Chau Doc (Fig.1F), providing high-resolution upstream forcing that captures both flood pulses and low-flow conditions. At the seaward boundary, tidal water levels were prescribed using eight primary astronomical constituents (M2, S2, N2, K2, K1, O1, P1, and Q1), supplemented by key shallow-water and long-period components (M4, MS4, MN4, MF, and MM) extracted from the TPXO 8.0 global tidal model. This combination supports representation of diurnal and semidiurnal tidal dynamics, as well as nonlinear tidal amplification within the delta's distributary channels. Along the offshore boundary, a Neumann-type tidal boundary formulation was used, in which amplitude and phase gradients between adjacent boundary support points were prescribed to minimize open-boundary artefacts and ensure stable propagation of tidal signals into the fluvial-deltaic network.

Supplementary Text 3: Salinity Forcing and Model Configuration

Salinity forcing was prescribed using freshwater conditions at the upstream boundaries and marine salinity at the offshore boundary. Upstream salinity was set to 0 psu, representing freshwater inflow from the Mekong River, while offshore salinity was set to 33 psu, representing marine conditions in the East Vietnam Sea (*30*). The salinity module was dynamically coupled with hydrodynamics, allowing simulated salinity distributions to respond to river discharge, tidal forcing, channel geometry, and mining-induced bathymetric changes. Although the model was implemented in a depth-averaged two-dimensional configuration, density effects associated with salinity gradients were represented using the Eckart equation of state. This enabled the model to capture basin-scale patterns of salinity intrusion and its response to changes in channel morphology, while recognizing that vertical stratification and salt-wedge structure cannot be fully resolved in a 2D framework. Salinity also influenced cohesive sediment behaviour through salinity-dependent settling, allowing enhanced settling of fine sediments under brackish conditions due to flocculation (*54*).

The depth-averaged setup was selected to balance process representation, computational efficiency, and data availability. While a fully three-dimensional model would better resolve vertical stratification, estuarine circulation, and near-bed salinity gradients (*46*), it would also require substantially greater computational resources and more extensive calibration data. For this study, the main objective was to quantify relative differences between mining and no-mining scenarios over a multi-year period. Because both scenarios used identical boundary forcing, parameter settings, and model configuration, the depth-averaged framework provides a suitable basis for assessing mining-driven changes in salinity intrusion, sediment redistribution, and delta-scale hydrodynamic response.

Supplementary Text 4: Sediment Transport and Morphodynamic Model Setup

To represent sediment dynamics in the VMD, a two-class sediment transport scheme was implemented in Delft3D-FM, separating cohesive clay/silt from non-cohesive sand. Long-term field measurements indicate that the upstream sediment supply is dominated by fine cohesive material, with clay and silt accounting for approximately 90−95% of total suspended sediment concentration and sand comprising only 5−10% (*19*, *60*, *61*). Based on these observations, a 95:5 clay-to-sand ratio was prescribed at the upstream boundaries of Tan Chau and Chau Doc, consistent with MRC suspended sediment observations (Fig.1F). Cohesive sediment transport was modelled using a depth-averaged approach with salinity-dependent settling velocity to represent flocculation under brackish conditions. Settling velocity increased from 0.0001 m/s in freshwater to 0.0005 m/s under saline conditions, reflecting enhanced aggregation and settling in estuarine environments (*30*, *62*). Clay erosion was represented using the Partheniades formulation and calibrated against observed seasonal suspended sediment concentrations. For the non-cohesive fraction, a median sand grain size (D50) of 0.25 mm was adopted based on sediment surveys in the VMD (*60*). This grain size is representative of distributary and delta-front bed material and is appropriate for simulating sand mobility and bedload transport. The dry bed density was set to 1650 kg/m^3. Sand settling velocity was computed internally by Delft3D-FM using the selected sand-transport formulation, typically yielding values of approximately 0.03−0.04 m/s, about two orders of magnitude higher than those used for cohesive sediments. Sand transport included both bedload and near-bed suspended-load components, with motion initiated when flow-induced bed shear stress exceeded the critical threshold for sediment mobility. The Van Rijn transport formulation was used to compute sand initiation, transport rates, and sediment fluxes.

Morphodynamic feedback were enabled by allowing bed updating during the simulation, applying slope-related sediment flux corrections, and using a critical Shields parameter to control sediment mobility. A morphological acceleration factor of one was applied, ensuring that bed evolution proceeded in real time rather than being artificially

accelerated. This configuration allowed dynamic exchange between the bed and water column and enabled simulation of erosion, deposition, and channel-bed adjustment over the multi-year model period. Direct measurements of bedload transport in the VMD are limited, making upstream bedload boundary specification uncertain. However, studies from the Mekong and other large rivers suggest that bedload generally represents a small fraction of the total sediment load, typically less than 5%, with suspended load dominating overall sediment delivery (*63*). Therefore, bedload input at the upstream boundaries was assumed to be negligible, while sand transport was allowed to develop dynamically within the model domain based on local hydraulics, bed shear stress, and sediment availability. This approach is consistent with the study's focus on basin-scale sediment redistribution and morphodynamic response rather than direct estimation of near-bed sediment fluxes at the upstream boundary.

Supplementary Text 5: Bathymetric Configuration and Data Integration

Bathymetric configuration was based primarily on high-resolution river-channel surveys conducted across the major distributary reaches of the VMD. Between 2017 and 2022, bathymetric surveys were collected using high-precision echo-sounding and acoustic instruments, including Teledyne Acoustic Doppler Current Profiler (ADCP), Hydrotrac II, and Humminbird HELIX 10 systems, providing depth measurements across river transects with reported vertical accuracy of approximately ±0.1−1% (*8*, *21*). For the present simulations, the pre-processed 2017 river-channel bathymetry from (*21*) and (*8*) was used as the initial bathymetric condition, with the period before December 2016 used for model spin-up and stabilization. Because the 2017 field surveys primarily covered the river-channel network, offshore and coastal bathymetry was supplemented using the GEBCO_2019 global bathymetric grid at approximately 450 m spatial resolution (https://www.gebco.net/data-products/gridded-bathymetry-data/arctic-ocean). High-resolution GEBCO gridded bathymetric products suitable for the offshore portion of the domain were available only for selected releases, including 2008, 2019, and 2023. Among these, GEBCO_2019 was selected because it was closest to the 2017 river-channel survey and simulation start period, whereas the 2008 grid was substantially older and the 2023 grid postdated the simulation period.

Although GEBCO_2019 postdates the start of the simulation by approximately two years, its use was considered appropriate for the offshore portion of the domain, where interannual bed-level changes are expected to be smaller than those in actively mined and morphodynamically dynamic river channels. The merged bathymetric surface therefore combined high-resolution field-survey data within the river network with global bathymetric data in offshore and coastal areas, ensuring continuous bed-elevation coverage across the full fluvial-estuarine-marine modelling domain. To reduce artefacts at the transition between surveyed river bathymetry and offshore GEBCO data, bathymetric surfaces were checked for consistency and smoothed where necessary before interpolation onto the flexible mesh. This step helped maintain stable bed-elevation gradients across the river-estuary-coastal transition while preserving the high-resolution channel morphology captured by the field surveys. During the simulation, bed levels were updated dynamically through the coupled sediment-transport and morphodynamic module, allowing erosion and deposition to evolve from the prescribed initial condition under observed hydrodynamic and sediment forcing. The suitability of the merged bathymetric configuration was evaluated through calibration and validation against independent observations of water levels, discharge, suspended sediment concentration, salinity, and bathymetric change during 2018−2020. The validation results showed no systematic model bias attributable to the merged bathymetric setup, supporting its use for long-term sediment transport and morphodynamic simulation in the VMD.

Supplementary Text 6: Initial Conditions and Two-Stage Simulation Setup

To ensure physically consistent start-up conditions, a two-stage simulation strategy was implemented. The first stage, from 1 November to 11 December 2016, served as a hydrodynamic, salinity, and sediment spin-up period to stabilize flow, salinity, and suspended-sediment fields before the main simulation. Uniform initial values were prescribed for water level (1.0 m), salinity (10 psu), horizontal eddy viscosity/diffusivity (160 m^2/s), and suspended-sediment concentration (0.01 kg/m^3). Bathymetry was derived from the 2017 survey dataset described above. The initial bed composition was represented using a single active bed layer containing two sediment fractions: cohesive clay and non-cohesive sand. The layer was initialized with sediment availability corresponding to 5 m of cohesive material and 10 m of sand, approximating the dominant clay-over-sand stratigraphic structure of the Mekong Delta. In the numerical model, this configuration allowed both sediment fractions to exchange dynamically with the water column according to local hydrodynamic conditions, sediment availability, and critical shear-stress thresholds.

This simplified bed configuration was designed to represent the dominant Holocene stratigraphy of the Mekong Delta, where a sand-rich basal unit is commonly overlain by muddy progradational deposits. Regional borehole evidence from the lower delta plain shows regressive stratigraphy in which prodelta and delta-plain muds overlie delta-front and mouth-bar sands, forming composite sequences approximately 10-25 m thick (*64*). This general structure is consistent with studies reporting sandy channel and mouth-bar deposits overlain by cohesive muds (*65*). Broader stratigraphic syntheses further identify a lower estuarine-to-marine sandy unit formed during post-glacial transgression, capped by muddy deltaic facies deposited during the Holocene highstand after approximately 8 ka (*66*). At a local scale, sediment cores from the abandoned Ba Lai distributary channel show rapid mud-dominated infilling, reaching up to 6.4 m

thickness, above older sandy channel deposits (*67*). Together, these observations support the use of a clay-over-sand conceptual structure as a generalized representation of bed-material availability in the deltaic substrate (*68*). During the spin-up, sediment exchange between cohesive and non-cohesive fractions allowed the initially prescribed bed composition to evolve toward a mixed active layer in morphodynamically active reaches. This configuration enabled cohesive-mud resuspension during high-flow periods and sand transport under stronger bed-shear conditions, while maintaining a simplified but physically motivated representation of bed-material availability. At the end of the spin-up period, simulated fields for water level, salinity, bed level, sediment thickness, and suspended-sediment concentration were exported and used as the initial conditions for the main simulation phase beginning on 12 December 2016. Morphodynamic updating was kept inactive until 1 January 2017 to further stabilize hydrodynamic and salinity conditions; after this date, bed evolution was allowed to respond dynamically to natural flow, tidal forcing, sediment transport, and sand-mining scenarios.

Supplementary Text 7: Tidal-Fluvial Decomposition Analysis

Discharge time series from 14 key cross-sections (CS01−CS14) were decomposed into fluvial and tidal components using a Godin-type three-pass 24-hour low-pass filter (*69*). The filter was applied to remove high-frequency tidal oscillations from the simulated discharge records, thereby isolating the low-frequency subtidal/fluvial discharge component associated with river forcing and seasonal flow variability. The tidal component was then calculated as the residual between the original discharge signal and the low-pass-filtered fluvial component. From these decomposed time series, we calculated a set of tidal-fluvial metrics, including mean river discharge, tidal discharge variability, tidal variance, and tidal dominance ratio. These metrics were used to quantify how sand-mining-induced bathymetric changes alter discharge redistribution, tidal propagation, and the relative contribution of tidal versus fluvial forcing across the deltaic channel network. The full suite of metrics, including their formulas, physical meaning, and relevance for assessing sand-mining impacts, is provided in table S2.

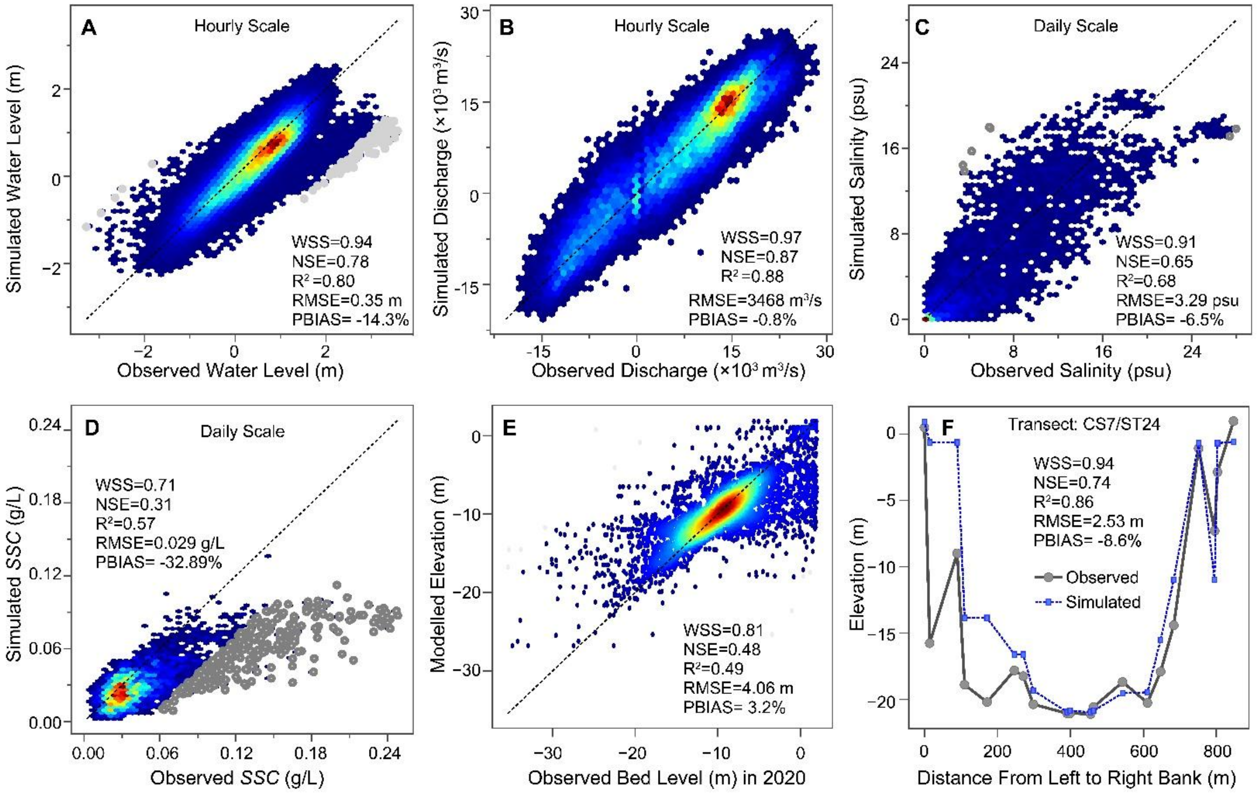

Fig. S1. Validation of the integrated Delft3D-FM model for the VMD from 2017 to 2021.
(A) Hourly water level; **(B)** hourly discharge;**(C**) daily salinity; **(D)** daily suspended sediment concentration (*SSC*); **(E)** bed level validation across more than 4,000 cross-sectional points collected in 2020; **(F)** example transect ST14, near Cross Section 7 (CS7) My Thuan bathymetry comparison for 2020. Scatter plots compare observed and simulated values with a 1:1 reference line, while panel f shows observed (blue) and simulated (black dashed) profiles. Across variables, the coupled model demonstrates consistently high predictive skill (WSS = 0.8−97), confirming its capability to reproduce tidal-fluvial water levels, discharge dynamics, salinity intrusion, sediment transport, and bathymetric patterns. These results provide strong confidence for subsequent scenarios of sand-mining impact simulations.

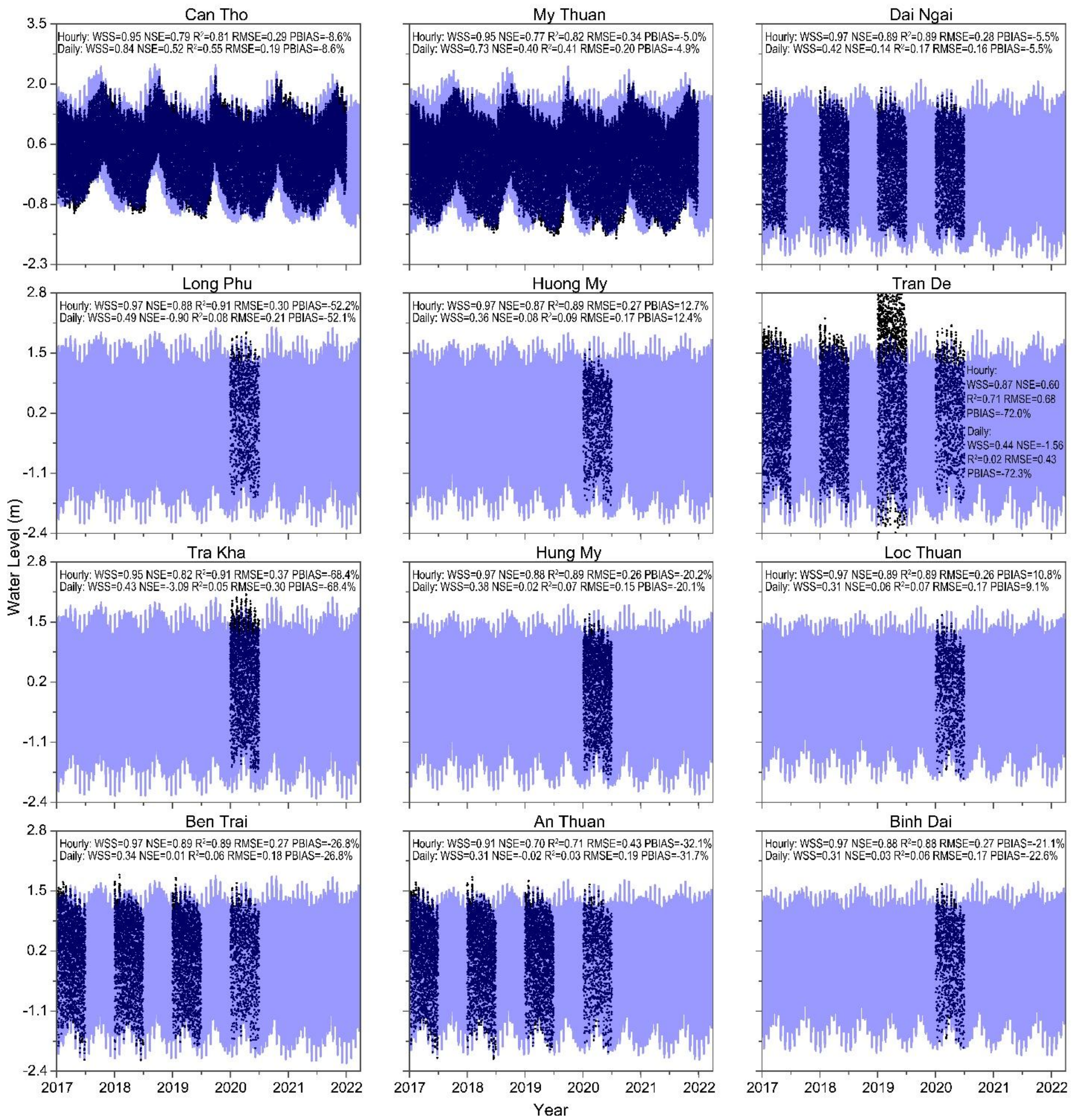

Fig. S2. Observed (dots) and simulated (blue line) water levels at representative VMD stations from 2017 to 2021. Model performance is summarized using hourly and daily skill metrics. The hourly simulations reproduce tidal water-level dynamics well, with high skill across most stations. When daily averaging is applied, the removal of tidal fluctuations reduces apparent model performance, especially in downstream reaches where tidal influence dominates. This indicates that short-term (hourly) evaluation better captures model fidelity to observed variability, while daily aggregation smooths tidal signals and highlights larger-scale biases.

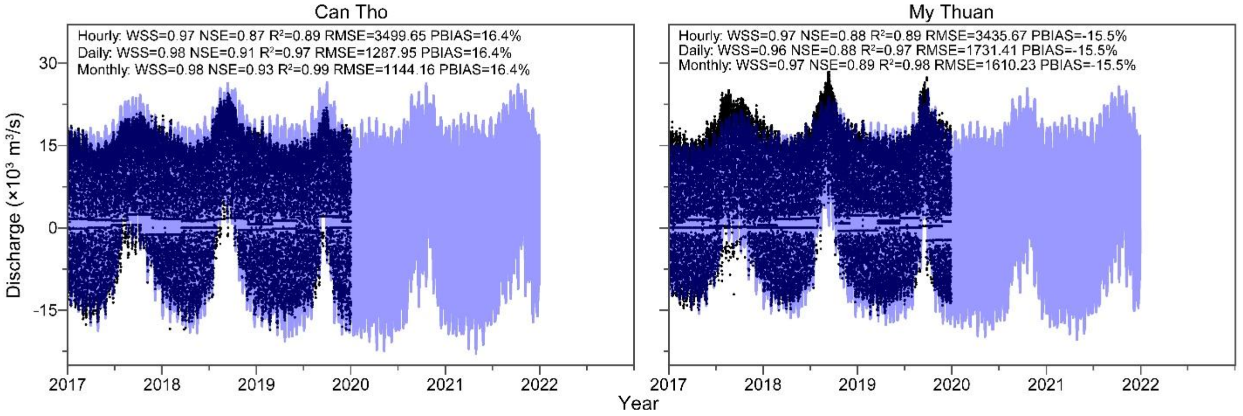

Fig. S3. Validation of simulated river discharge at Can Tho and My Thuan stations.
Observed (black) and simulated (blue) discharge time series are shown with model performance metrics at hourly, daily, and monthly scales. The model accurately reproduces both the amplitude and timing of flood pulses, monsoon surges, and dry-season low flows. Higher skill at daily and monthly scales reflects the removal of tidal reversals and short-term phase offsets, allowing the model's strong agreement with the net river-flow dynamics to emerge, unlike water-level validation, where averaging removes the dominant tidal signal and reduces apparent skill.

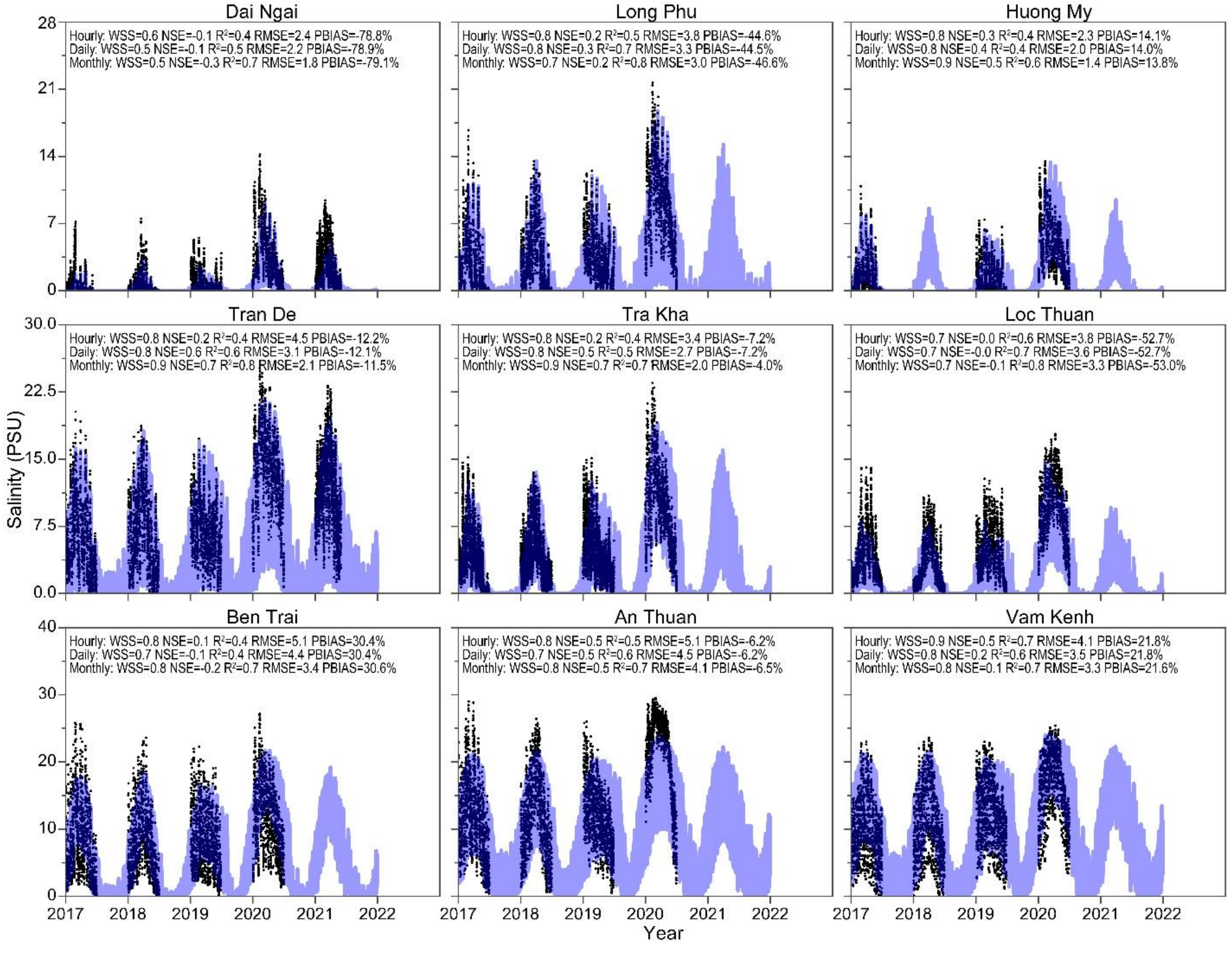

Fig. S4. Validation of simulated salinity at estuarine stations from 2017 to 2021.
Time series of 2-hourly observed (black) and simulated (blue) salinity are shown at multiple key locations across the VMD. Model performance metrics are presented for hourly, daily, and monthly timescales. While short-term (hourly) variability is influenced by tidal phase differences and mixing processes, temporal averaging at daily and monthly scales reduces tidal noise and highlights the model's ability to reproduce longer-term salinity dynamics. Improved skill at coarser timescales therefore reflects the model's accuracy in capturing mean salt intrusion and seasonal variability rather than short-term fluctuations.

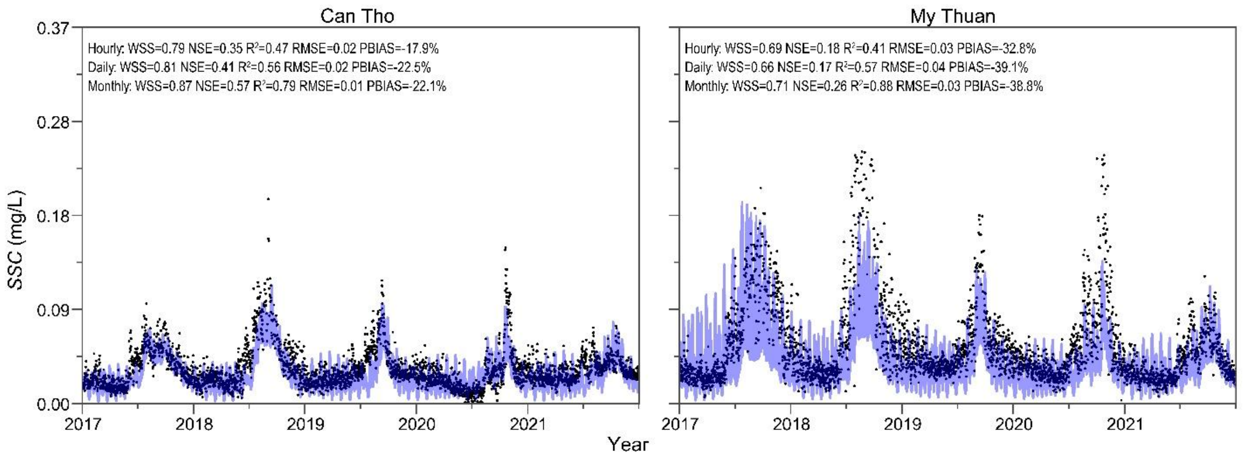

Fig. S5. Validation of simulated suspended sediment concentration (*SSC*) at Can Tho and My Thuan stations from 2017 to 2021.

Observed (black) and simulated (blue) *SSC* time series are shown with hourly, daily, and monthly performance metrics. Model skill improves at daily and monthly scales because temporal averaging reduces high-frequency tidal and turbulent variability and aligns the model output with the observational sampling frequency, field *SSC* measurements were typically collected only twice per day (at high and low tide). The enhanced agreement therefore reflects both a closer temporal match and the model's ability to capture mean sediment transport dynamics rather than instantaneous fluctuations.

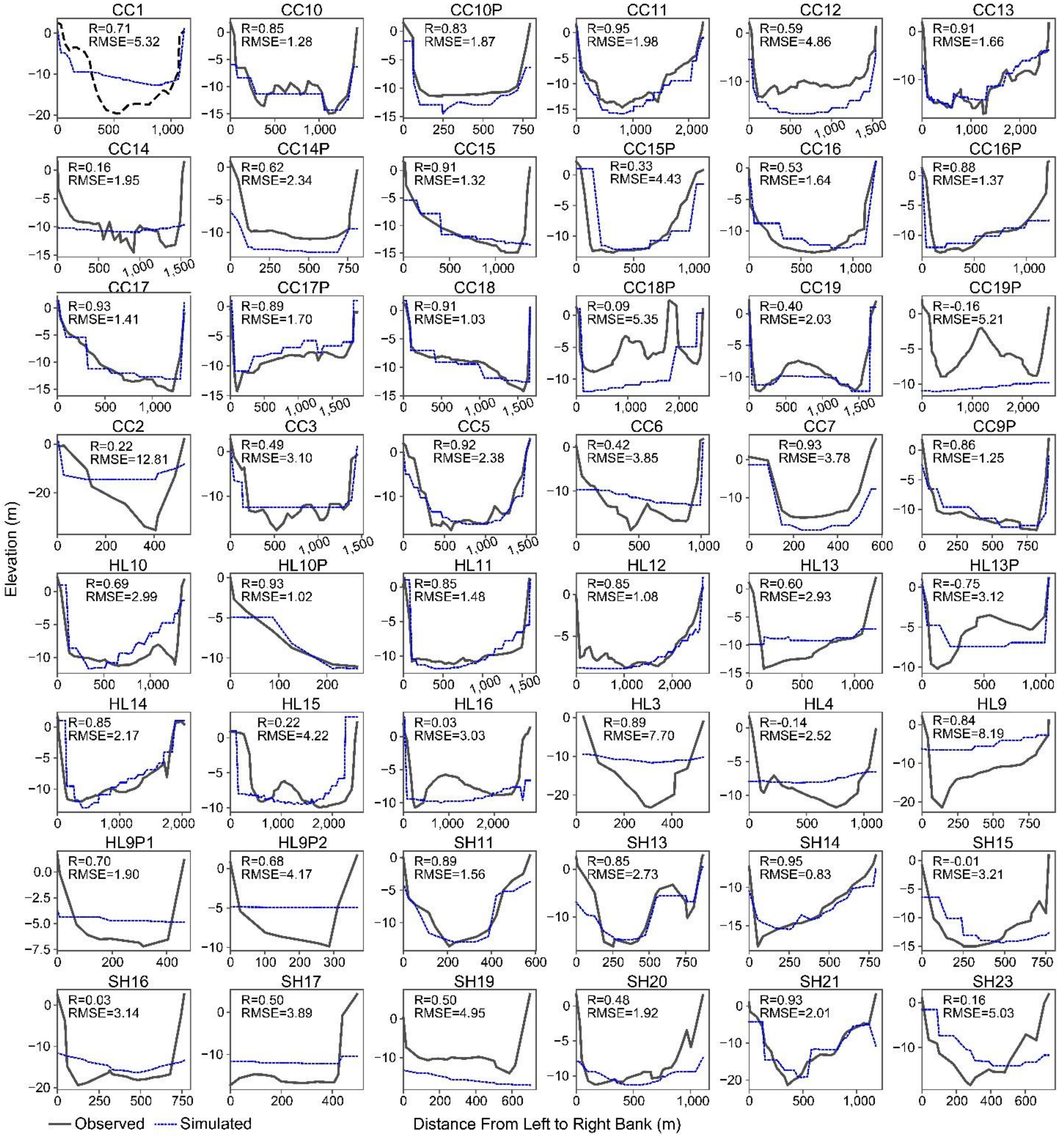

CC1
R=0.71
RMSE=5.32
CC10
R=0.85
RMSE=1.28
CC10P
R=0.83
RMSE=1.87
CC11
R=0.95
RMSE=1.98
CC12
R=0.59
RMSE=4.86
CC13
R=0.91
RMSE=1.66
CC14
R=0.16
RMSE=1.95
CC14P
R=0.62
RMSE=2.34
CC15
R=0.91
RMSE=1.32
CC15P
R=0.33
RMSE=4.43
CC16
R=0.53
RMSE=1.64
CC16P
R=0.88
RMSE=1.37
CC17
R=0.93
RMSE=1.41
CC17P
R=0.89
RMSE=1.70
CC18
R=0.91
RMSE=1.03
CC18P
R=0.09
RMSE=5.35
CC19
R=0.40
RMSE=2.03
CC19P
R=-0.16
RMSE=5.21
CC2
R=0.22
RMSE=12.81
CC3
R=0.49
RMSE=3.10
CC5
R=0.92
RMSE=2.38
CC6
R=0.42
RMSE=3.85
CC7
R=0.93
RMSE=3.78
CC9P
R=0.86
RMSE=1.25
HL10
R=0.69
RMSE=2.99
HL10P
R=0.93
RMSE=1.02
HL11
R=0.85
RMSE=1.48
HL12
R=0.85
RMSE=1.08
HL13
R=0.60
RMSE=2.93
HL13P
R=-0.75
RMSE=3.12
HL14
R=0.85
RMSE=2.17
HL15
R=0.22
RMSE=4.22
HL16
R=0.03
RMSE=3.03
HL3
R=0.89
RMSE=7.70
HL4
R=-0.14
RMSE=2.52
HL9
R=0.84
RMSE=8.19
HL9P1
R=0.70
RMSE=1.90
HL9P2
R=0.68
RMSE=4.17
SH11
R=0.89
RMSE=1.56
SH13
R=0.85
RMSE=2.73
SH14
R=0.95
RMSE=0.83
SH15
R=-0.01
RMSE=3.21
SH16
R=0.03
RMSE=3.14
SH17
R=0.50
RMSE=3.89
SH19
R=0.50
RMSE=4.95
SH20
R=0.48
RMSE=1.92
SH21
R=0.93
RMSE=2.01
SH23
R=0.16
RMSE=5.03
Elevation (m)
Distance From Left to Right Bank (m)
Observed
Simulated

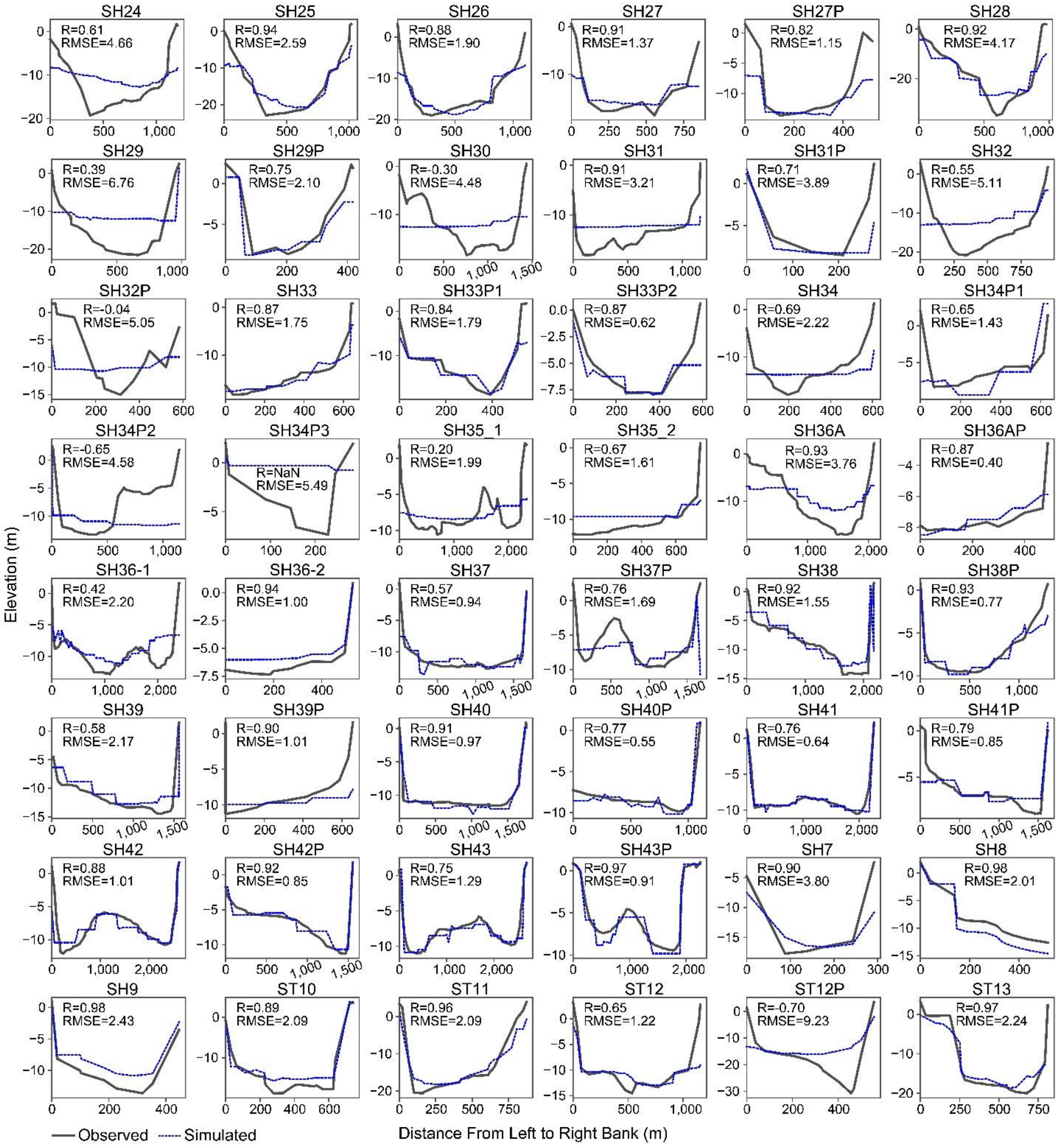
SH24
R=0.61
RMSE=4.66
SH25
R=0.94
RMSE=2.59
SH26
R=0.88
RMSE=1.90
SH27
R=0.91
RMSE=1.37
SH27P
R=0.82
RMSE=1.15
SH28
R=0.92
RMSE=4.17
SH29
R=0.39
RMSE=6.76
SH29P
R=0.75
RMSE=2.10
SH30
R=-0.30
RMSE=4.48
SH31
R=0.91
RMSE=3.21
SH31P
R=0.71
RMSE=3.89
SH32
R=0.55
RMSE=5.11
SH32P
R=-0.04
RMSE=5.05
SH33
R=0.87
RMSE=1.75
SH33P1
R=0.84
RMSE=1.79
SH33P2
R=0.87
RMSE=0.62
SH34
R=0.69
RMSE=2.22
SH34P1
R=0.65
RMSE=1.43
SH34P2
R=-0.65
RMSE=4.58
SH34P3
R=NaN
RMSE=5.49
SH35_1
R=0.20
RMSE=1.99
SH35_2
R=0.67
RMSE=1.61
SH36A
R=0.93
RMSE=3.76
SH36AP
R=0.87
RMSE=0.40
SH36-1
R=0.42
RMSE=2.20
SH36-2
R=0.94
RMSE=1.00
SH37
R=0.57
RMSE=0.94
SH37P
R=0.76
RMSE=1.69
SH38
R=0.92
RMSE=1.55
SH38P
R=0.93
RMSE=0.77
SH39
R=0.58
RMSE=2.17
SH39P
R=0.90
RMSE=1.01
SH40
R=0.91
RMSE=0.97
SH40P
R=0.77
RMSE=0.55
SH41
R=0.76
RMSE=0.64
SH41P
R=0.79
RMSE=0.85
SH42
R=0.88
RMSE=1.01
SH42P
R=0.92
RMSE=0.85
SH43
R=0.75
RMSE=1.29
SH43P
R=0.97
RMSE=0.91
SH7
R=0.90
RMSE=3.80
SH8
R=0.98
RMSE=2.01
SH9
R=0.98
RMSE=2.43
ST10
R=0.89
RMSE=2.09
ST11
R=0.96
RMSE=2.09
ST12
R=0.65
RMSE=1.22
ST12P
R=-0.70
RMSE=9.23
ST13
R=0.97
RMSE=2.24
Elevation (m)
Distance From Left to Right Bank (m)
Observed
Simulated

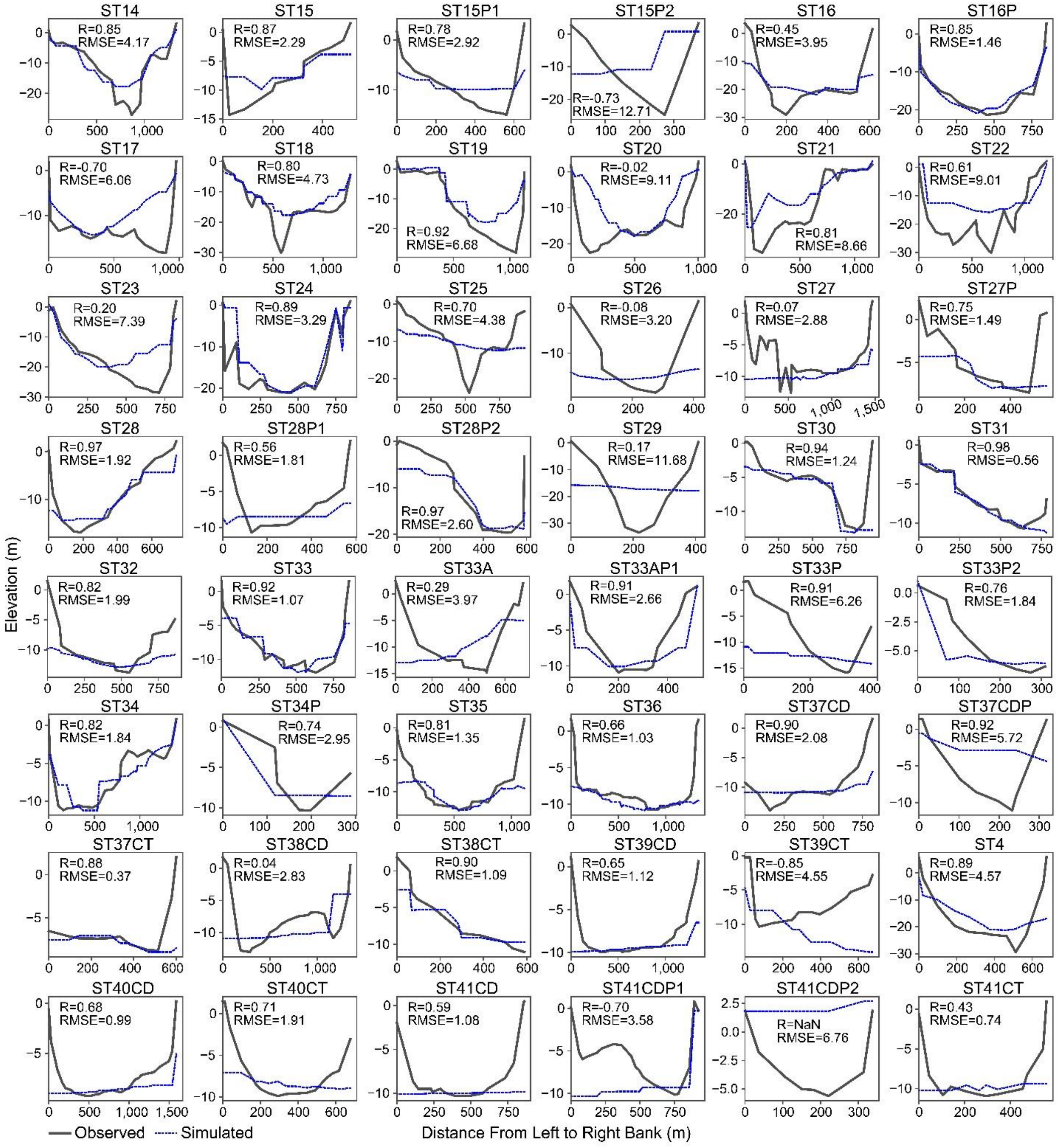
ST14
R=0.85
RMSE=4.17
ST15
R=0.87
RMSE=2.29
ST15P1
R=0.78
RMSE=2.92
ST15P2
R=-0.73
RMSE=12.71
ST16
R=0.45
RMSE=3.95
ST16P
R=0.85
RMSE=1.46
ST17
R=-0.70
RMSE=6.06
ST18
R=0.80
RMSE=4.73
ST19
R=0.92
RMSE=6.68
ST20
R=-0.02
RMSE=9.11
ST21
R=0.81
RMSE=8.66
ST22
R=0.61
RMSE=9.01
ST23
R=0.20
RMSE=7.39
ST24
R=0.89
RMSE=3.29
ST25
R=0.70
RMSE=4.38
ST26
R=-0.08
RMSE=3.20
ST27
R=0.07
RMSE=2.88
ST27P
R=0.75
RMSE=1.49
ST28
R=0.97
RMSE=1.92
ST28P1
R=0.56
RMSE=1.81
ST28P2
R=0.97
RMSE=2.60
ST29
R=0.17
RMSE=11.68
ST30
R=0.94
RMSE=1.24
ST31
R=0.98
RMSE=0.56
ST32
R=0.82
RMSE=1.99
ST33
R=0.92
RMSE=1.07
ST33A
R=0.29
RMSE=3.97
ST33AP1
R=0.91
RMSE=2.66
ST33P
R=0.91
RMSE=6.26
ST33P2
R=0.76
RMSE=1.84
ST34
R=0.82
RMSE=1.84
ST34P
R=0.74
RMSE=2.95
ST35
R=0.81
RMSE=1.35
ST36
R=0.66
RMSE=1.03
ST37CD
R=0.90
RMSE=2.08
ST37CDP
R=0.92
RMSE=5.72
ST37CT
R=0.88
RMSE=0.37
ST38CD
R=0.04
RMSE=2.83
ST38CT
R=0.90
RMSE=1.09
ST39CD
R=0.65
RMSE=1.12
ST39CT
R=-0.85
RMSE=4.55
ST4
R=0.89
RMSE=4.57
ST40CD
R=0.68
RMSE=0.99
ST40CT
R=0.71
RMSE=1.91
ST41CD
R=0.59
RMSE=1.08
ST41CDP1
R=-0.70
RMSE=3.58
ST41CDP2
R=NaN
RMSE=6.76
ST41CT
R=0.43
RMSE=0.74
Elevation (m)
Distance From Left to Right Bank (m)
Observed
Simulated

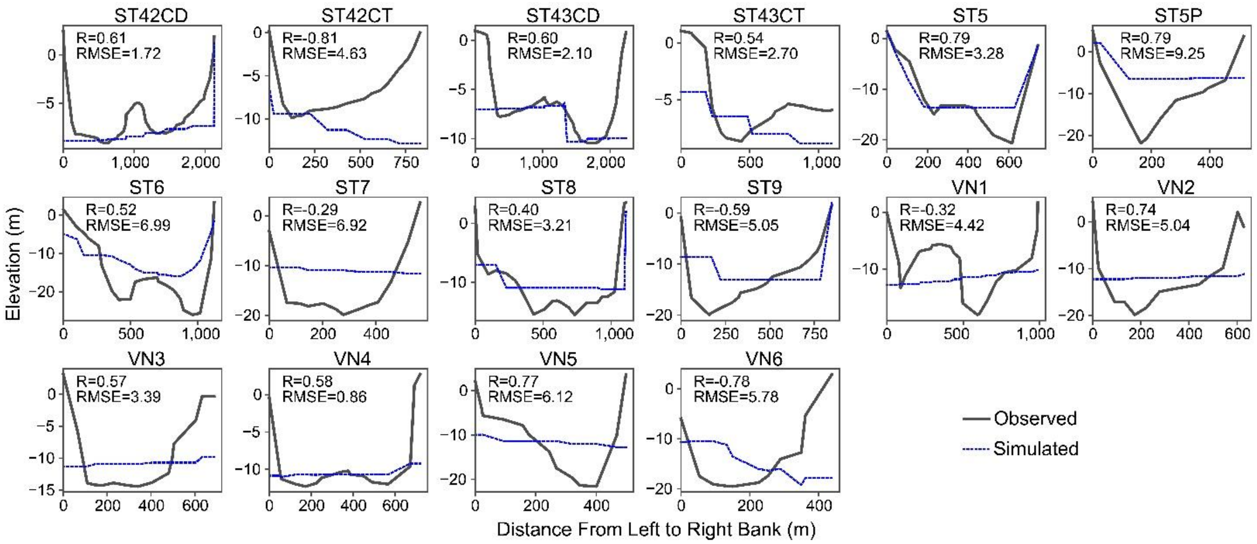

Fig. S6. Validation of simulated bathymetric profiles across 180 cross-sections in the VMD in 2020.
Black lines denote observed elevations, and blue dashed lines represent simulated results. Transect-level performance metrics are shown for each profile. The model reproduced bed morphology well across most reaches, with spatially consistent performance and minor deviations confined to narrow tributaries or areas with limited bathymetric coverage. Across 4,296 cross-sectional points, 38.7% of simulated depths were within ±1 m and 57.6% within ±2 m of observations, while larger deviations (> 6 m) accounted for only 14.9%. These discrepancies reflect localized data and interpolation uncertainties rather than systematic model bias.

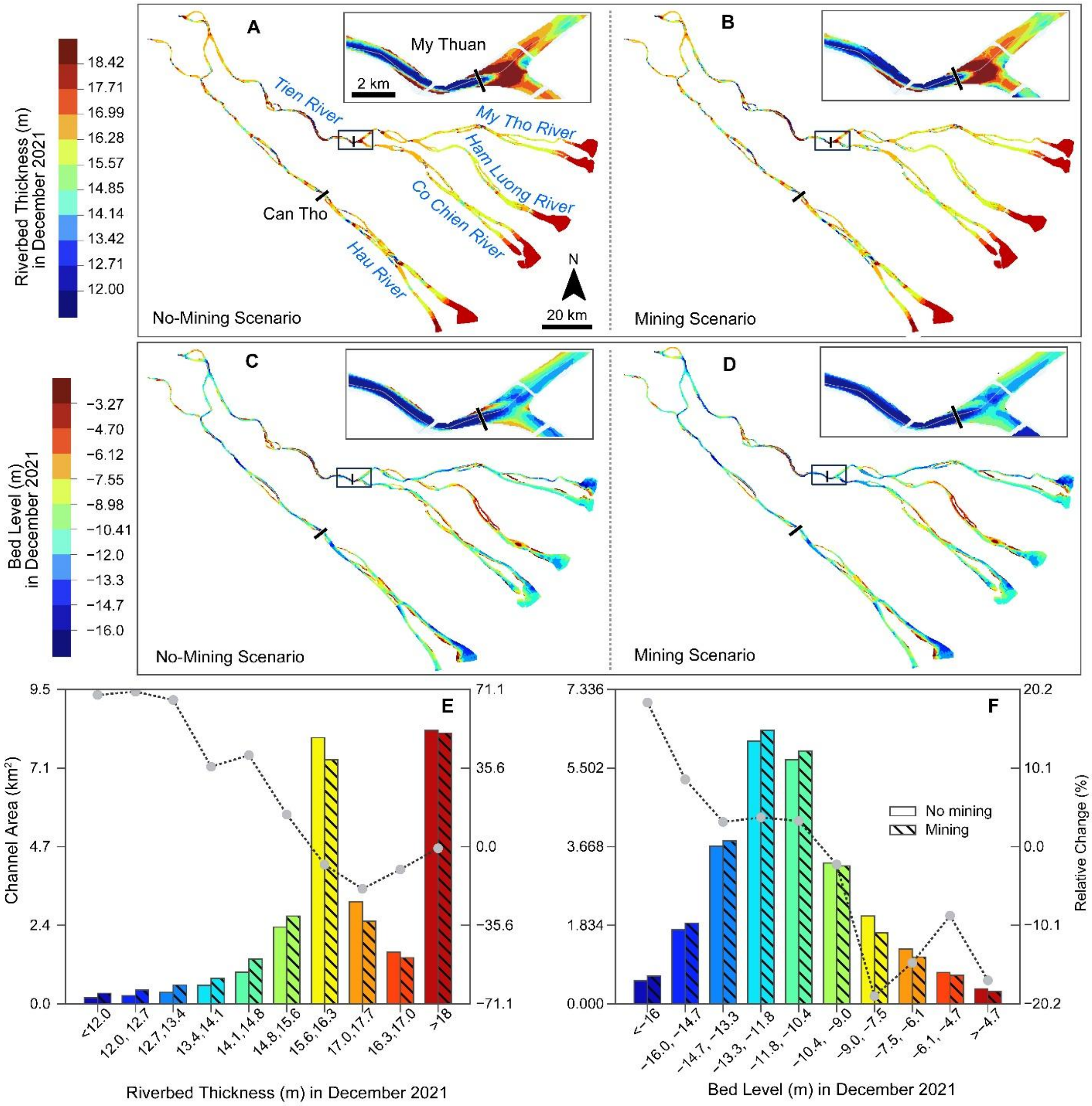

Fig. S7. Spatial comparison of modelled riverbed thickness, and bed elevation in the VMD under contrasting sand-mining scenarios from 2017 to 2021.

(A and B) show riverbed thickness in December 2021, and **(C and D)** riverbed level relative to mean sea level. **(E and F)** quantify changes in channel area across value classes, showing an increase in the thinnest sediment class (< 13.4 m; 69%), expansion of deeper bed-level zones (< -16 m; 18%). Collectively, the results confirm that sand mining substantially enhances erosion and channel deepening while reducing the delta's sediment storage capacity.

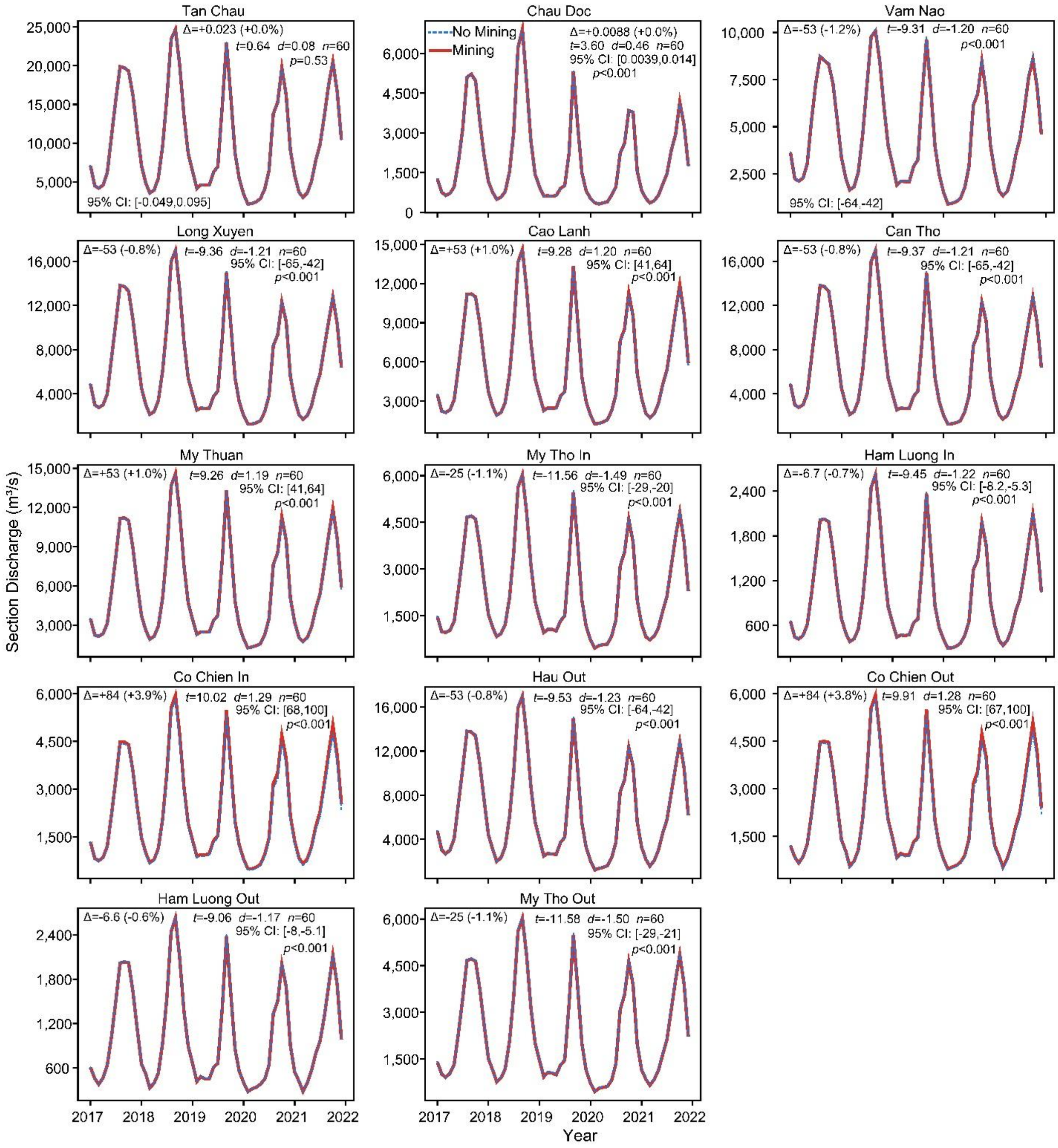

Fig. S8. Cross-sectional discharge (from 2017 to 2021) under no-mining and mining scenarios.
Monthly mean discharge time series for fourteen representative cross-sections along the Tien-Hau distributary system. Red and blue lines indicate mining and no-mining simulations, respectively. The results show spatially variable discharge responses to sand extraction, with mid-delta reaches (e.g., Vam Nao, Long Xuyen, Can Tho) exhibiting statistically significant decreases of ≈ 0.8−1.2 % (p < 0.001), while upstream stations (Tan Chau, Chau Doc) remain largely unchanged. Inset statistics show the mean difference between mining and no-mining simulations (Δ), relative change compared with no-mining conditions (%), paired t-test statistic (t), Cohen's d effect size (d), sample size (n, monthly values), 95% confidence interval of the mean difference, and p-value.

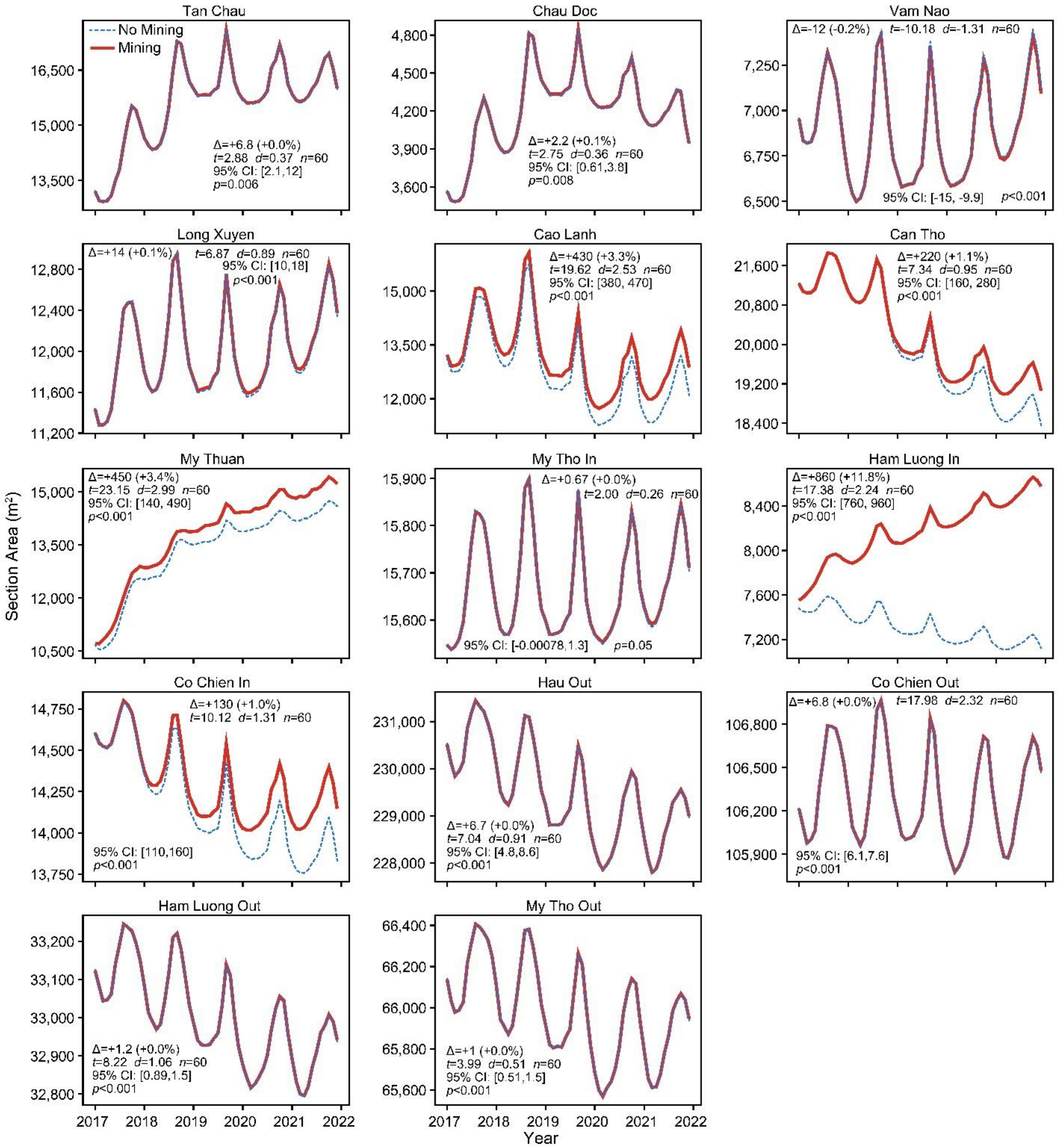

Fig. S9. Wetted cross-sectional area (from 2017 to 2021) under no-mining and mining scenarios.
Time-series comparison of total wetted area for fourteen sections, demonstrating widespread channel enlargement under the mining scenario. Area increases are most pronounced at Ham Luong In (11.5 %), followed by moderate yet significant expansions at Cao Lanh (3.6 %) and Can Tho (1.3 %) ($p < 0.001$). These changes indicate channel widening and bank retreat induced by sediment removal, which collectively increase conveyance capacity but reduce flow velocity. Inset statistics are defined as in Fig. S8.

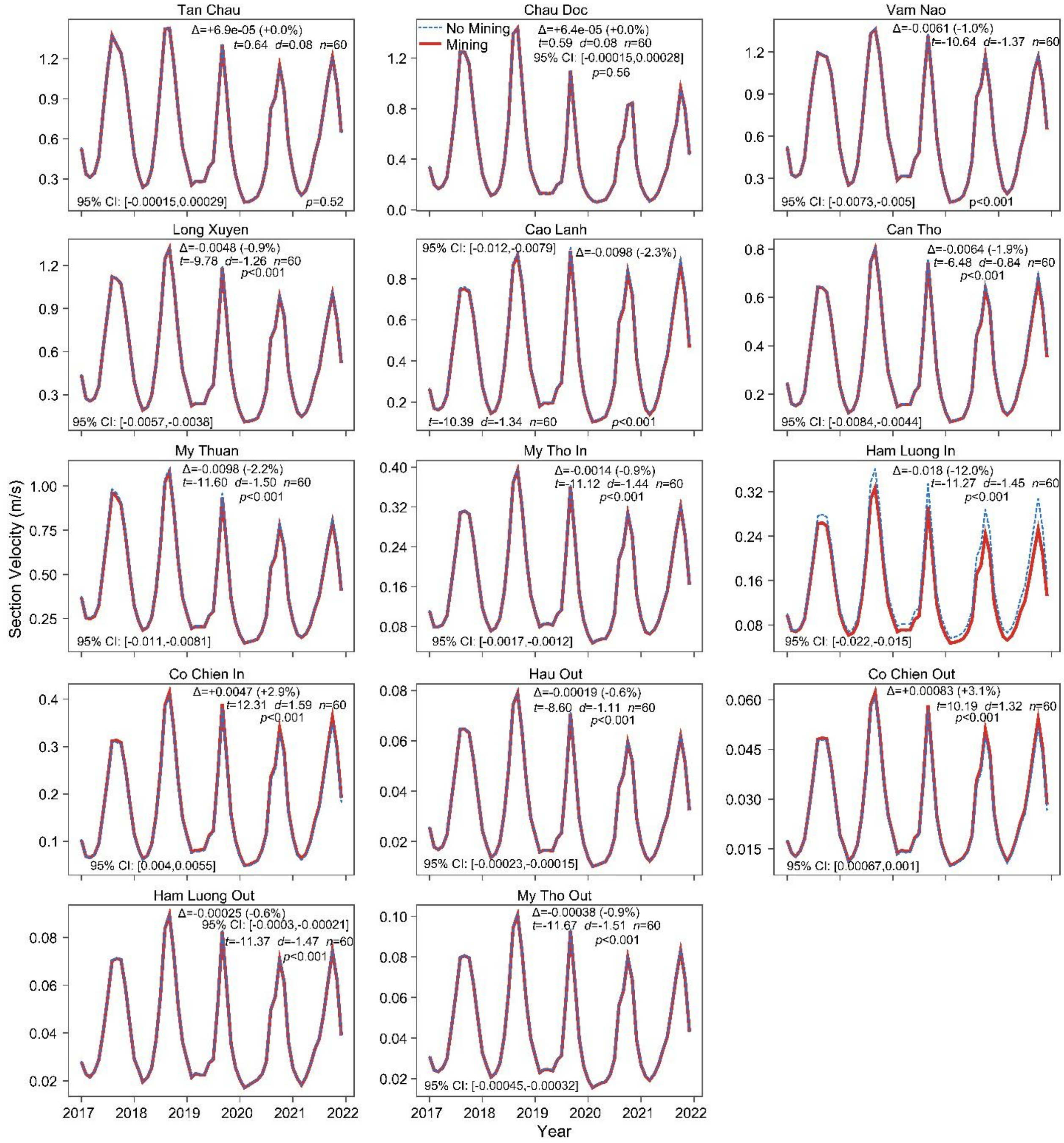

Fig. S10. Cross-sectional velocity (from 2017 to 2021) under no-mining and mining scenarios.
Temporal evolution of section-mean velocity at the same fourteen stations. Mining scenarios generally yield lower velocities across mid- and downstream reaches, reflecting reduced flow energy within deepened or widened channels. Notable declines occur at Can Tho (-1.9 %) and Ham Luong In (-11.9 %, $p < 0.001$), consistent with reduced hydraulic gradients and enhanced cross-sectional area. Upstream reaches (Tan Chau, Chau Doc) show negligible change. These patterns confirm that sediment extraction alters both flow competence and channel hydraulics. Inset statistics are defined as in Fig. S8. Inset statistics are defined as in Fig. S8.

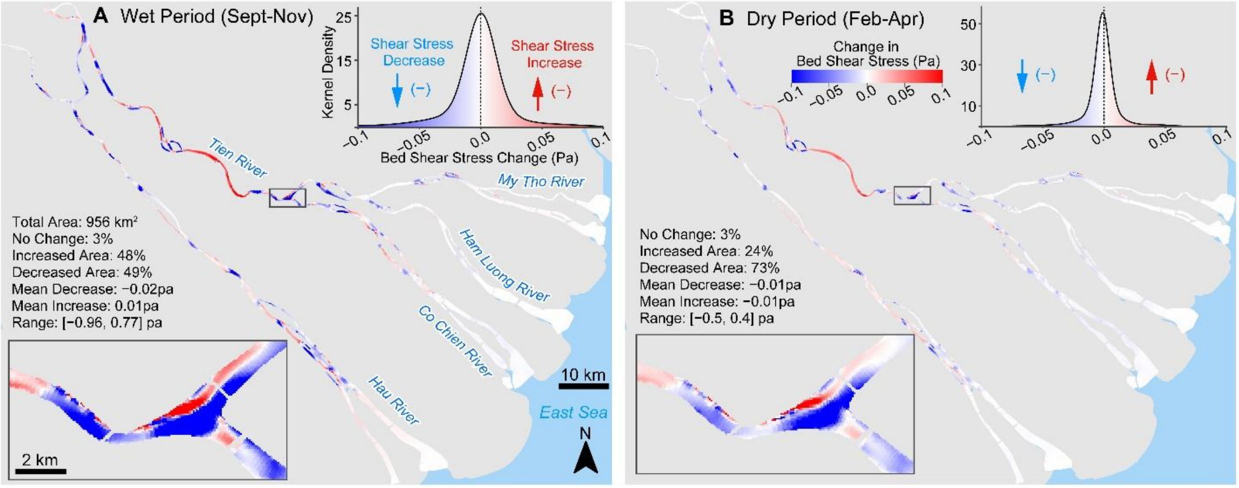

Fig. S11. Seasonal changes in bed shear stress ($\Delta\tau_{(b)} = \tau_{(b,\ mining)} - \tau_{(b,\ no\text{-}mining)}$) across the VMD.
(A) Wet period (September−November) and **(B)** dry period (February−April) maps show the spatial difference in depth-averaged bed shear stress between mining and no-mining simulations. Red areas denote shear-stress increase (+), and blue areas denote reduction (-). Insets (top) display the probability distribution of $\Delta\tau_{(b)}$, while lower insets provide a zoomed example near the My Thuan bifurcation. During the wet period, moderate shear-stress increases occur mainly along upper distributaries due to higher discharge, whereas the dry period shows widespread decreases across mid- and lower reaches, especially along the Hau and Ham Luong Rivers, caused by mining-induced deepening and reduced bed friction. Localized red zones indicate persistent stress amplification near constricted bifurcations where flow convergence remains strong. Overall, the results demonstrate that sand mining weakens near-bed hydraulic stress over most of the delta while intensifying local erosion potential at morphologically sensitive nodes.

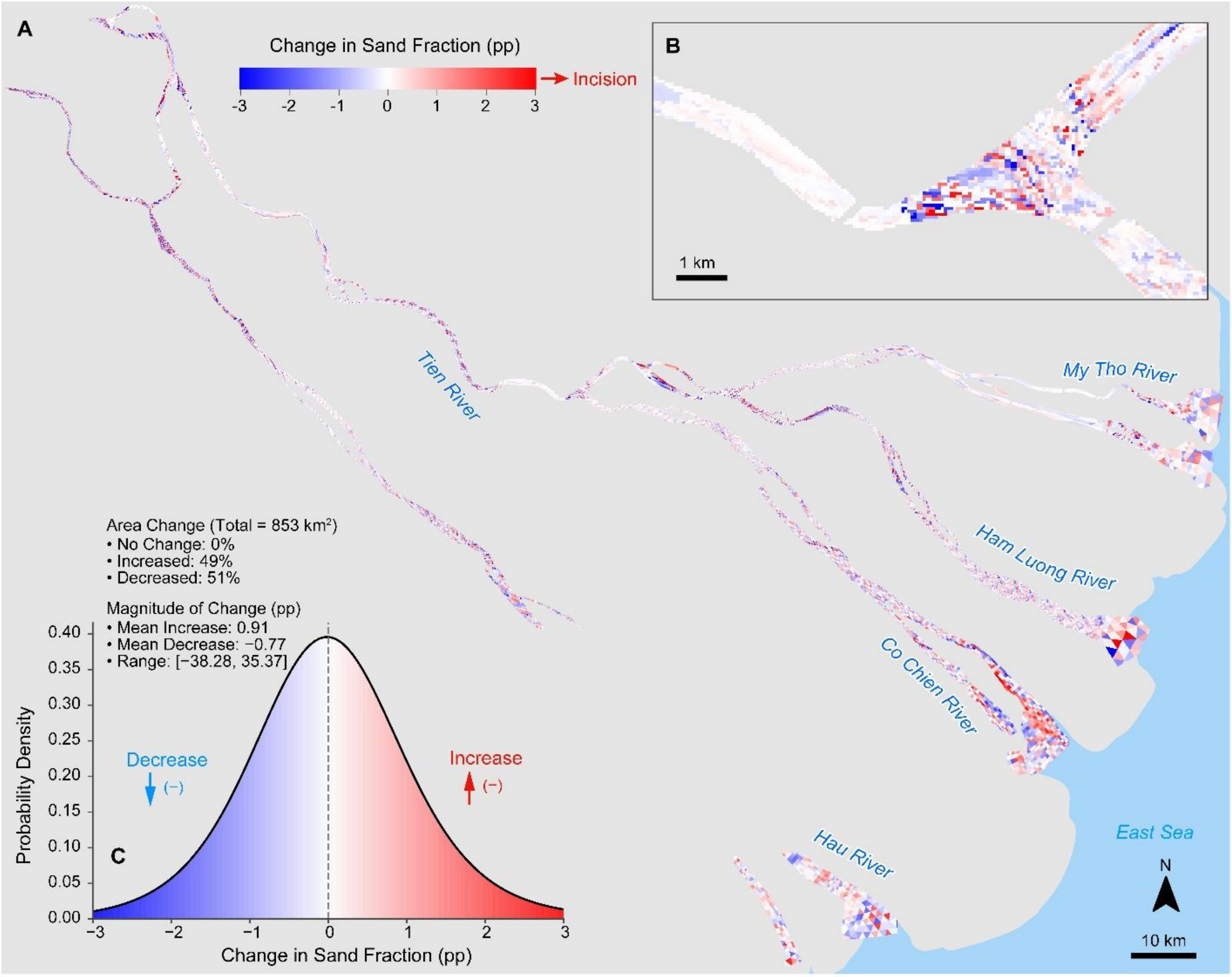

Fig. S12. Bed-material composition response to sand mining in the VMD.
Spatial distribution of changes in bed sand fraction (mining - no-mining) across the deltaic distributaries. Red areas denote sand enrichment and blue areas denote depletion, illustrating alternating zones of incision and deposition concentrated around active mining reaches and channel confluences. The inset highlights localized bed coarsening at the confluence of the Tien-Hau system. The lower panel shows the probability density of sand-fraction change, indicating a near-balanced response: ≈ 50% of the bed area experienced change, with mean increases of 0.9 pp and decreases of -0.8 pp. Extreme values reach ±3 pp, reflecting localized sand exposure and fine-sediment removal. Together, these results show that sand mining reorganizes bed texture gradients through selective erosion and deposition rather than uniform fining.

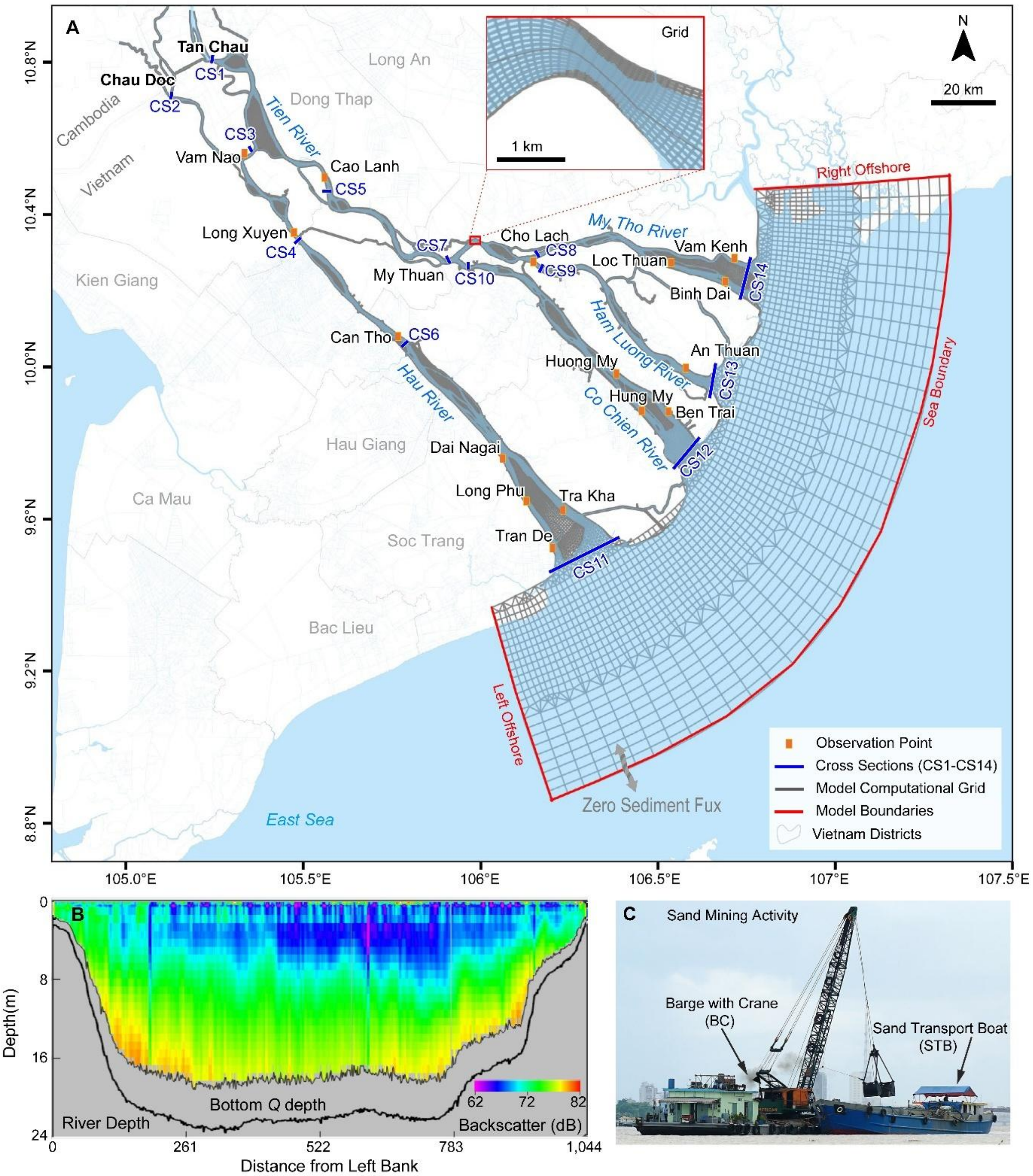

Fig. S13. Study domain, model setup, and key datasets for the VMD.

(A) The Delft3D-FM flexible mesh computational grid, showing upstream boundaries (Tan Chau, Chau Doc), the coastal boundary, hydraulic monitoring stations (orange rectangles), and analysis cross-sections (blue lines, CS1−CS14). **(B)** Exemplary Acoustic Doppler Current Profiler (ADCP) transect (Hau River, near Can Tho) showing riverbed bathymetry and backscatter intensity, used for model setup and calibration. **(C)** In-situ photograph of sand mining activity, showing a Barge with Crane (BC) and a Sand Transport Boat (STB). Photo credit: Edward Park.

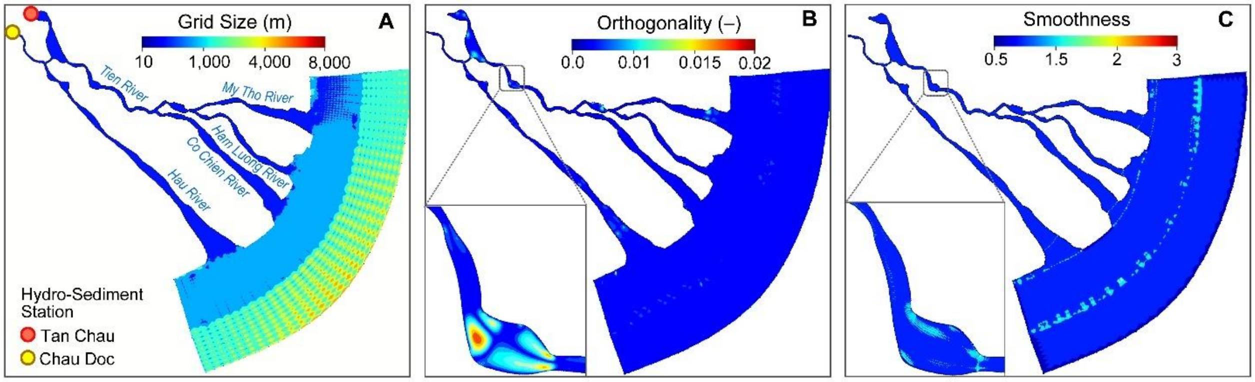

Fig. S14. Model domain and boundary forcing data for the Mekong Delta hydro-sedimentological model.
(A) Computational grid showing spatial resolution (edge lengths) and boundary locations. **(B)** Grid orthogonality metric indicating numerical accuracy. **(C)** Grid smoothness coefficient.

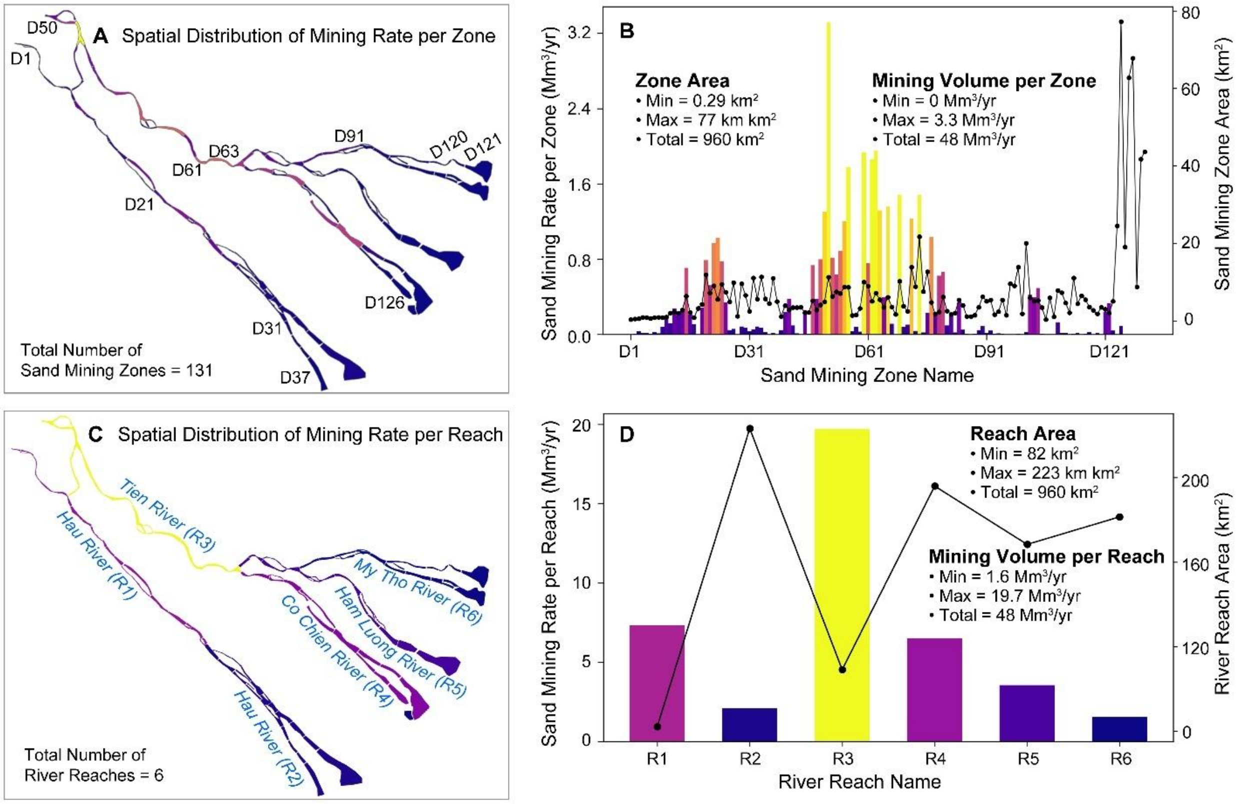

Fig. S15. Spatial distribution and extraction volumes of sand mining in the Vietnamese Mekong Delta.
Delineation of 131 sand mining zones along the main channels, based on reported activity. **(A)** Annual extraction per zone (bars; Mm^3/yr) and **(B)** corresponding zone area (line; km^2), showing that a small number of zones account for the majority of total removal. **(C)** Aggregation of zones into six major river reaches (R1−R6) for comparative assessment. **(D)** Annual extraction per reach (bars; Mm^3/yr) and corresponding reach area (line; km^2), highlighting that Reach 3 contributes nearly half of total extraction.

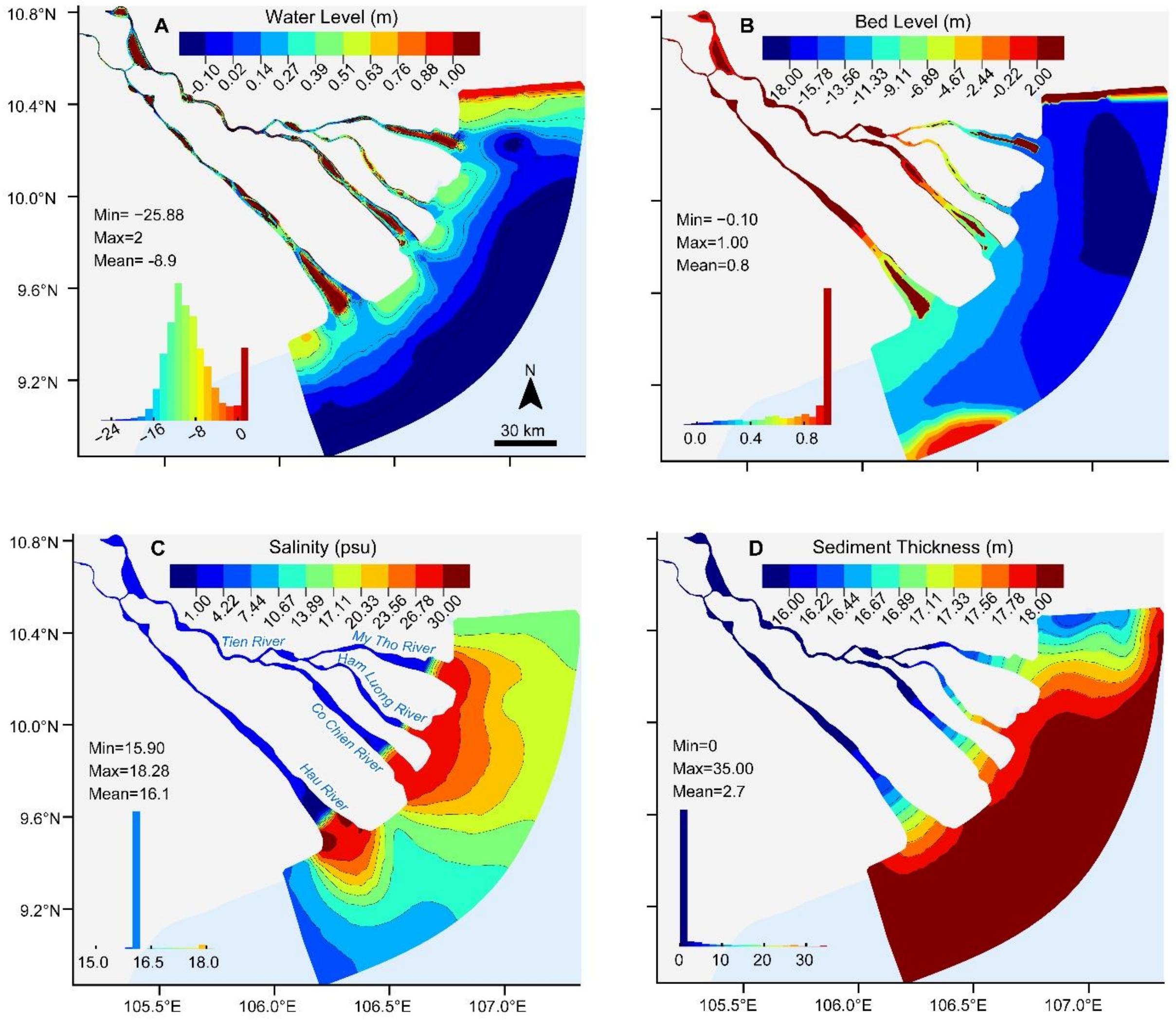

Fig. S16. Model state following the initial spin-up phase, serving as the baseline for the main simulation. Spatial distributions at the end of the first initialization stage (11 December 2016): **(A)** bed elevation (m), **(B)** water level (m), **(C)** sediment thickness (m), and **(D)** salinity (psu). Insets show the corresponding value histograms for the entire domain. These fields, produced after a six-week model spin-up from 1 November 2016, provide the physically consistent initial condition used to start the final 20-day stabilization run (12−31 December 2016), preceding the main production simulation from 1 January 2017.

Table S1. Major hydropower dams commissioned on the Mekong River system from 2017 to 2021.
This table summarizes major hydropower dams commissioned along the Mekong River and its key tributaries between 2017 and 2022. The period marked the rapid completion of several mainstream and tributary projects, particularly in China (Lancang section) and Laos, that substantially increased regional hydropower capacity. Collectively, these projects contributed more than ~7 GW of new installed capacity and have significantly altered downstream discharge, sediment continuity, and seasonal flow regimes within the Mekong Basin.

Dam Name	Country	River Name	Commission Year	Installed Capacity (MW)
Dahuaqiao	China	Mekong (Lancang)	2018	920
Wunonglong	China	Mekong (Lancang)	2018	990
Lidi	China	Mekong (Lancang)	2019	420
Xayaburi	Laos	Mekong	2019	1,285
Don Sahong	Laos	Mekong	2020	240
Tuoba	China	Mekong (Lancang)	2024*	1,400
Lower Sesan 2	Cambodia	Sesan River (Mekong Tributary)	2018	400
Nam Ou 2, 5, 6, 7	Laos	Nam Ou River (Mekong Tributary)	2017–2020	~600 (combined)
Xekaman 1	Laos	Xekaman River (Mekong Tributary)	2018	322
Xekaman 3	Laos	Xekaman River (Mekong Tributary)	2019	250
Nam Theun 1	Laos	Nam Theun River (Mekong Tributary)	2021	650

Note: *Tuoba Dam began partial operation in 2024 but was planned under the 2017−2022 cascade expansion (*36*).

Table S2. Summary of river-tide decomposition metrics across 14 representative cross-sections in the VMD under mining and no-mining scenarios.
ΔQ_{river} reports the absolute and percentage change in mean freshwater discharge, while ΔTDV and ΔE_{tide} quantify the corresponding changes in spring-neap tidal variability and tidal-discharge variance. ΔD_{tide} represents the change in the tidal dominance ratio, indicating the shift in the relative contribution of tidal variance to total discharge variability. The results show consistent discharge redistribution, with negative ΔQ_{river} (~-53 m^3/yr) along the Hau branch and positive ΔQ_{river} (~53 to 85 m^3/yr) along the Tien-Co Chien system. Increases in tidal variability and tidal variance are strongest at mid-delta stations (Can Tho, My Thuan, Cao Lanh, and Co Chien In), where channel deepening enhances tidal penetration and mixing, yielding ΔD_{tide} values of ~0.005−0.009. Upstream stations (Tan Chau, Chau Doc) and outer estuarine outlets (Hau Out, Ham Luong Out, My Tho Out) show minimal changes, consistent with low sensitivity to local incision. Overall, these diagnostics indicate that sand-mining-induced deepening strengthens tidal influence in the mid-delta while maintaining relatively stable river-tide conditions near boundaries.

Station	ΔQ_{river}(m^3/s)	ΔQ_{river} (%)	ΔTDV	ΔTDV (%)	ΔE_{tide}	ΔE_{tide} (%)	ΔD_{tide}
Tan Chau	0.02	0.00	0.42	0.02	0.04	0.00	0.000060
Chau Doc	0.01	0.00	0.05	0.01	0.01	0.00	0.000026
Vam Nao	-52.97	-1.18	-0.28	-0.01	-0.35	-1.18	0.003942
Can Tho	-53.32	-0.83	177.52	1.96	3.93	-0.83	0.007688
My Thuan	52.70	0.95	240.71	2.99	6.24	0.95	0.006527
Cao Lanh	52.85	0.96	204.54	3.17	6.41	0.96	0.008668
Long Xuyen	-53.24	-0.82	110.39	1.50	2.79	-0.82	0.007486
My Tho In	-24.80	-1.07	18.12	0.32	0.63	-1.07	0.001245
Co Chien In	84.53	3.86	164.27	4.76	10.10	3.86	0.005234
Ham Luong In	-6.77	-0.66	18.63	1.30	2.80	-0.66	0.005904
Hau Out	-53.17	-0.82	-0.86	0.00	0.00	-0.82	0.000250
Co Chien Out	83.87	3.84	-66.04	-0.26	-0.51	3.84	-0.000236
Ham Luong Out	-6.60	-0.64	-17.94	-0.16	-0.32	-0.64	0.000039
My Tho Out	-24.93	-1.07	10.80	0.06	0.12	-1.07	0.000108

Table S3. Statistical performance metrics used for model calibration and validation.

Formulas, valid ranges, and definitions are provided for each metric. The Willmott Skill Score (WSS) and Nash–Sutcliffe Efficiency (NSE) assess overall predictive agreement and model efficiency relative to observed means. The Coefficient of Determination (R^2) quantifies explained variance, while the Root Mean Square Error (RMSE) measures the magnitude of model deviation. Percent Bias (PBIAS) indicates the average tendency of the model to over- or under-predict observations. Together, these complementary indicators evaluate both accuracy and reliability of hydrodynamic, salinity, and sediment-transport simulations.

**Name**	**Formula**	**Definition**
Willmott Skill Score (WSS)	$WSS = 1 - \frac{\sum_{i=1}^{n}(S_i - O_i)^2}{\sum_{i=1}^{n}(\lvert S_i - \underline{O}\rvert + \lvert O_i - \underline{O}\rvert)^2}$	Quantifies the degree of agreement between simulated and observed values by comparing the mean squared error to a potential error term that accounts for both the magnitude and direction of deviations from the observed mean. Unlike correlation-based metrics, the Willmott Skill Score (also called the Index of Agreement) is sensitive to differences in both bias and variability, yet less affected by proportional errors or outliers. Values close to 1 indicate strong agreement and high predictive reliability, while values near 0 indicate poor model performance.
Nash-Sutcliffe Efficiency (NSE)	$NSE = 1 - \frac{\sum_{i=1}^{n}(S_i - O_i)^2}{\sum_{i=1}^{n}(0_i - \underline{O})^2}$	Measures the predictive skill of the model relative to the mean of observations. NSE = 1 represents a perfect match, NSE = 0 indicates performance equivalent to using the mean of observations, and NSE <0 implies performance worse than the mean.
Coefficient of Determination (R^2)	$R^2 = \left(\frac{\sum_{i=1}^{n}(O_i - \underline{O})(S_i - \underline{S})}{\sqrt{\sum_{i=1}^{n}(O_i - \underline{O})^2} \times \sqrt{\sum_{i=1}^{n}(S_i - \underline{S})^2}}\right)^2$	Quantifies how much of the observed variance is captured by the model. Widely used for numerical model calibration
Root Mean Square Error (RMSE)	$RMSE = \sqrt{\frac{1}{n}\sum_{i=1}^{n}(S_i - O_i)^2}$	Measures the average magnitude of deviation between simulated and observed values, giving greater weight to larger errors. Lower RMSE indicates higher model accuracy.
Percent Bias (PBIAS)	$PBIAS = \frac{\sum_{i=1}^{n}(S_i - O_i)}{\sum_{i=1}^{n} O_i}$	Indicates the model's average tendency to overestimate (negative PBIAS) or underestimate (positive PBIAS) observations. A value of 0 represents perfect agreement.

Note: In the formulas, O_i represents the observed value at instance *i*, S_i is the simulated value at instance *i*, O_i denotes the mean of observed values, $\underline{S}$ denotes the mean of simulated values, and *n* is the total number of observations.

Table S4. Diagnostic metrics used to quantify river-tide decomposition and hydrodynamic responses to channel deepening.

The table summarizes the discharge-based metrics applied to separate river and tidal components using a Godin-type low-pass filter, quantify spring-neap tidal variability, and evaluate how morphological change alters tidal forcing and river-tide balance. Each metric includes its mathematical definition and the corresponding physical meaning used to interpret hydrodynamic responses under no-mining and mining scenarios.

Metric Name	Formula / Definition	Physical Meaning
River Discharge Component (Q_{river})	Three-pass 24-hour running mean of total discharge: $Q_{river} = mean_{24h} (mean_{24h} (mean_{24h} (Q_{total})))$	Low-frequency freshwater discharge from upstream, representing background river flow after tidal signals are removed. Useful for detecting how morphological changes alter mean river conveyance.
Tidal Discharge Component (Q_{tidal})	$Q_{tidal} = Q_{total} .- Q_{river}$	High-frequency oscillatory discharge associated with tidal inflow and outflow around the mean river flow. Indicates how changes in channel geometry influence tidal signal strength.
Tidal Discharge Variability (TDV)	$TDV = \sigma_{15d} (Q_{tidal})$	Short-term (spring-neap) variability of the tidal discharge component. Sensitive to frictional and geometric modifications that enhance tidal penetration.
Tidal Discharge Variance (E_{tide})	$E_{tide} = Var (Q_{tidal})$	System-wide variance of tidal discharge, reflecting the overall magnitude of tidal oscillations. Summarizes the integrated effect of morphological changes on tidal forcing.
Tidal Dominance *Ratio* (D_{tide})	$D_{tide} = E_{tide} / E_{total}$	Fraction of total discharge variability controlled by tidal motion rather than river flow. Indicates shifts in the river-tide balance along the channel.
Discharge Change (ΔQ_{river} %)	$100 \times (Q_{river, mining} .- Q_{river, nomining})/Q_{river, nomining}$	Percent change in mean freshwater discharge between simulations. Reveals how altered channel geometry affects hydraulic resistance and flow conveyance.
Tidal Discharge Variability Change (ΔTDV %)	$100 \times (\mathrm{TDV}_{tide,mining} - TDV_{tide,nomining})/TDV_{tidenomining}$	Percent change in spring-neap tidal variability. Highlights system response to reductions in friction or bathymetric deepening.
Tidal Variance Change (ΔE_{tide} %)	$100 \times (E_{tide,mining} - E_{tide,nomining})/ E_{tide,nomining}$	Percent change in the variance of tidal discharge. Captures how channel modification alters the magnitude of tidal fluctuations.
Tidal Dominance Change ($\Delta \mathrm{D}_{\mathrm{tide}}$)	$D_{tide,mining}, - D_{tide,nomining}$	Change in the contribution of tidal variance to total discharge variability. Indicates transitions from river-dominated to more tide-dominated conditions.

REFERENCES

1. United Nations Environment Programme (UNEP), "Sand and sustainability: Finding new solutions for environmental governance of global sand resources" (GRID-Geneva, 2019).

2. United Nations Environment Programme (UNEP), "Sand and sustainability: 10 Strategic recommendations to avert a crisis" (GRID-Geneva, 2022).

3. D. D. Tran, E. Park, J. Wang, H. H. Loc, J. Lee, S. Zhan, S. A. Kantoush, Environmental pressures on livelihood transformation in the Vietnamese Mekong Delta: Implications and adaptive pathways. *J. Environ. Manage.* **377**, 124597 (2025).

4. W. J. Ang, E. Park, Y. Pokhrel, D. D. Tran, H. H. Loc, Dams in the Mekong: A comprehensive database, spatiotemporal distribution, and hydropower potentials. *Earth Syst. Sci. Data* **16**, 1209–1228 (2024).

5. R. J. P. Schmitt, Z. Rubin, G. M. Kondolf, Losing ground—Scenarios of land loss as consequence of shifting sediment budgets in the Mekong Delta. *Geomorphology* **294**, 58–69 (2017).

6. S. D. X. Chua, Y. Yang, G. M. Kondolf, C. Oeurng, T. Sok, S. Zhang, X. Lu, Can restoring water and sediment fluxes across a mega-dam cascade alleviate a sinking river delta? *Sci. Adv.* **10**, eadn9731 (2024).

7. J. Best, Anthropogenic stresses on the world's big rivers. *Nat. Geosci.* **12**, 7–21 (2019).

8. D. V. Binh, S. Kantoush, T. Sumi, Changes to long-term discharge and sediment loads in the Vietnamese Mekong Delta caused by upstream dams. *Geomorphology* **353**, 107011 (2020).

9. G. M. Kondolf, R. J. P. Schmitt, P. A. Carling, M. Goichot, M. Keskinen, M. E. Arias, S. Bizzi, A. Castelletti, T. A. Cochrane, S. E. Darby, M. Kummu, P. S. J. Minderhoud, D. Nguyen, H. T. Nguyen, N. T. Nguyen, C. Oeurng, J. Opperman, Z. Rubin, D. C. San, S. Schmeier, T. Wild, Save the Mekong Delta from drowning. *Science* **376**, 583–585 (2022).

10. C. R. Gruel, E. Park, A. D. Switzer, S. Kumar, H. H. Loc, S. Kantoush, D. V. Binh, L. Feng, New systematically measured sand mining budget for the Mekong Delta reveals rising trends and significant volume underestimations. *Int. J. Appl. Earth Obs. Geoinf.* **108**, 102736 (2022).

11. S. Kumar, E. Park, D. D. Tran, J. Wang, H. H. Loc, L. Feng, S. A. Kantoush, D. V. Binh, D. Li, A. D. Switzer, A deep learning framework to map riverbed sand mining budgets in large tropical deltas. *GIsci. Remote Sens.* **61**, 2285178 (2024).

12. A. Torres, M. U. Simoni, J. K. Keiding, D. B. Müller, S. O. S. E. zu Ermgassen, J. Liu, J. A. G. Jaeger, M. Winter, E. F. Lambin, Sustainability of the global sand system in the Anthropocene. *One Earth* **4**, 639–650 (2021).

13. H. H. Loc, E. Park, The salinization of the Mekong Delta: Major drivers, coping strategies, and new hopes from ecosystem-based approaches. *Curr. Opin. Environ. Sustain.* **77**, 101584 (2025).

14. J. A. Duberstein, K. W. Krauss, M. J. Baldwin, S. T. Allen, W. H. Conner, J. S. Salter, M. Miloshis, Small gradients in salinity have large effects on stand water use in freshwater wetland forests. *For. Ecol. Manage.* **473**, 118308 (2020).

15. S. L. C. Xin, E. Park, D. D. Tran, K. W. Yuen, J. Wang, Landscape and social disruption from sand mining and mining-related activities: A case from the Vietnamese Mekong Delta. *Ann. Am. Assoc. Geogr.* **114**, 1968–1984 (2024).

16. K. W. Yuen, E. Park, D. D. Tran, H. H. Loc, L. Feng, J. Wang, C. R. Gruel, A. D. Switzer, Extent of illegal sand mining in the Mekong Delta. *Commun. Earth Environ.* **5**, 31 (2024).

17. E. J. Anthony, G. Brunier, M. Besset, M. Goichot, P. Dussouillez, V. L. Nguyen, Linking rapid erosion of the Mekong River delta to human activities. *Sci. Rep.* **5**, 14745 (2015).

18. G. Brunier, E. J. Anthony, M. Goichot, M. Provansal, P. Dussouillez, Recent morphological changes in the Mekong and Bassac river channels, Mekong delta: The marked impact of river-bed mining and implications for delta destabilisation. *Geomorphology* **224**, 177–191 (2014).

19. C. Jordan, J. Tiede, O. Lojek, J. Visscher, H. Apel, H. Q. Nguyen, C. N. X. Quang, T. Schlurmann, Sand mining in the Mekong Delta revisited–Current scales of local sediment deficits. *Sci. Rep.* **9**, 17823 (2019).

20. G. M. Kondolf, Z. K. Rubin, J. T. Minear, Dams on the Mekong: Cumulative sediment starvation. *Water Resour. Res.* **50**, 5158–5169 (2014).

21. R. Y. S. Lau, E. Park, D. D. Tran, J. Wang, Recent intensification of riverbed mining in the Mekong Delta revealed by extensive bathymetric surveying. *J. Hydrol.* **626**, 130174 (2023).

22. L. N. Anh, D. D. Tran, N. Thong, C. T. Van, D. H. Vinh, N. H. Au, E. Park, Drastic variations in estuarine morphodynamics in Southern Vietnam: Investigating riverbed sand mining impact through hydrodynamic modelling and field controls. *J. Hydrol.* **608**, 127572 (2022).

23. L. Koehnken, M. S. Rintoul, M. Goichot, D. Tickner, A. C. Loftus, M. C. Acreman, Impacts of riverine sand mining on freshwater ecosystems: A review of the scientific evidence and guidance for future research. *River Res. Appl.* **36**, 362–370 (2020).

24. T. T. Kim, N. T. M. Huong, N. D. Q. Huy, P. A. Tai, S. Hong, T. M. Quan, N. T. Bay, W. K. Jeong, N. K. Phung, Assessment of the impact of sand mining on bottom morphology in the Mekong River in An Giang province, Vietnam, using a hydro-morphological model with GPU computing. *Water* **12**, 2912 (2020).

25. N. A. Nguyen, V. H. Pham, A. B. Nguyen, T. P. T. Giang, V. T. Le, C. H. Nguyen, T. T. Thai, Spatiotemporal dynamics of suspended sediment in coastal Mekong Delta: A hydrodynamic modelling approach under tropical monsoon climate. *Sci. Rep.* **15**, 5851 (2025).

26. L. Q. Quan, C. R. Hackney, G. Vasilopoulos, T. Coulthard, N. N. Hung, S. E. Darby, D. R. Parsons, Sand-mining-driven reduction in Tonle Sap Lake's critical flood pulse. *Nat. Sustain.* **8**, 1455–1466 (2025).

27. E. Park, Sand mining in the Mekong Delta: Extent and compounded impacts. *Sci. Total Environ.* **924**, 171620 (2024).

28. G. Vasilopoulos, Q. L. Quan, D. R. Parsons, S. E. Darby, V. P. D. Tri, N. N. Hung, I. D. Haigh, H. E. Voepel, A. P. Nicholas, R. Aalto, Establishing sustainable sediment budgets is critical for climate-resilient mega-deltas. *Environ. Res. Lett.* **16**, 064089 (2021).

29. B. Q. Nguyen, S. A. Kantoush, T. Sumi, Quantifying the consequences of unsustainable sand mining and cascade dams on aspects in a tropical river basin. *Sci. Rep.* **14**, 1178 (2024).

30. X. T. Le, Q. T. Vo, J. Reyns, P. V. Song, T. A. Duong, D. D. Thanh, D. Roelvink, Sediment transport and morphodynamical modeling on the estuaries and coastal zone of the Vietnamese Mekong Delta. *Cont. Shelf Res.* **186**, 64–76 (2019).

31. D. V. Binh, S. A. Kantoush, R. Ata, P. Tassi, T. V. Nguyen, J. Lepesqueur, K. E. K. Abderrezzak, S. E. Bourban, Q. H. Nguyen, D. N. L. Phuong, L. V. Trung, D. A. Tran, T. Letrung, T. Sumi, Hydrodynamics, sediment transport, and morphodynamics in the Vietnamese Mekong Delta: Field study and numerical modelling. *Geomorphology* **413**, 108368 (2022).

32. A. Gasparotto, A. P. Nicholas, G. H. S. Smith, A. Daham, "Modelling the impact of sand extraction from large rivers" in *River Flow 2022*, A. M. Ferreira da Silva, C. Rennie, S. Gaskin, J. Lacey, B. MacVicar, Eds. (CRC Press, 2024), pp. 463–471.

33. M. T. Vu, C. Luu, D. Q. Bui, Q. H. Vu, M. Q. Pham, Simulation of hydrodynamic changes and salinity intrusion in the lower Vietnamese Mekong Delta under climate change-induced sea level rise and upstream river discharge. *Reg. Stud. Mar. Sci.* **78**, 103749 (2024).

34. T. T. Kim, T. N. Q. Nga, N. D. Q. Huy, N. K. Phung, H. C. Hoai, N. T. Bay, The impact of sand mining on the bed morphology of the Tien River, Mekong Delta, Vietnam. *Environ. Earth Sci.* **84**, 108 (2025).

35. T. N. Thanh, H. H. Van, H. V. Minh, V. P. D. Tri, Salinity intrusion trends under the impacts of upstream discharge and sea level rise along the Co Chien River and Hau River in the Vietnamese Mekong Delta. *Climate* **11**, 66 (2023).

36. B. Eyler, R. Kwan, "All dams map of the Mekong basin" (Stimson Center, 2024); www.stimson.org/2024/all-dams-map-of-the-mekong-basin/.

37. M. Bendixen, L. L. Iversen, J. Best, D. M. Franks, C. R. Hackney, E. M. Latrubesse, L. S. Tusting, Sand, gravel, and UN Sustainable Development Goals: Conflicts, synergies, and pathways forward. *One Earth* **4**, 1095–1111 (2021).

38. P. Zhang, Q. Yang, H. Liu, Z. Dai, J. Lin, X. Zhang, H. Cai, Tracking hydrodynamic variation in a tide-dominant estuary over the past half-century. *J. Hydrol.* **663**, 134191 (2025).

39. B. Barman, B. Kumar, A. K. Sarma, Dynamic characterization of the migration of a mining pit in an alluvial channel. *Int. J. Sediment Res.* **34**, 155–165 (2019).

40. R. M. Frings, Downstream fining in large sand-bed rivers. *Earth Sci. Rev.* **87**, 39–60 (2008).

41. E. Park, D. D. Tran, P. S. J. Minderhoud, R. Clarke, F. K. S. Chan, Practical paths to halt elevation loss in Vietnamese Mekong Delta. *Geogr. Sustainability* **6**, 100335 (2025).

42. P. S. J. Minderhoud, "Modelling Mekong Delta subsidence, challenges and how to improve quantifications" in *The 4th Asia Pacific Meeting on Near Surface Geoscience & Engineering* (European Association of Geoscientists & Engineers, 2021), vol. 2021, pp. 1–7; www.earthdoc.org/content/papers/10.3997/2214-4609.202177047.

43. P. S. J. Minderhoud, L. Coumou, G. Erkens, H. Middelkoop, E. Stouthamer, Mekong delta much lower than previously assumed in sea-level rise impact assessments. *Nat. Commun.* **10**, 3847 (2019).

44. I. N. Monioudi, M. I. Vousdoukas, A. Giardino, A. Stocchino, L. Mentaschi, L. Feyen, Impacts of sea level rise and adaptation across Asia and the Pacific. *Sci. Rep.* **15**, 35742 (2025).

45. J. Bauer, F. Dörr, H. T. D. Vu, A. Schenk, H. V. Tran, V. C. Pham, N. Börsig, R. van der Linden, N. H. Nguyen, E. Eiche, S. Norra, Seawater intrusion in river delta systems. Inter-annual dynamics and drivers of salinity variations in the southern Mekong Delta, Vietnam. *J. Hydrol.* **661**, 133745 (2025).

46. S. Eslami, P. Hoekstra, N. N. Trung, S. A. Kantoush, D. V. Binh, D. D. Dung, T. T. Quang, M. van der Vegt, Tidal amplification and salt intrusion in the Mekong Delta driven by anthropogenic sediment starvation. *Sci. Rep.* **9**, 18746 (2019).

47. P. N. Duc, T. T. Duc, G. P. Van, H. N. Van, T. T. Minh, Predicting salinity levels in the Mekong delta (Viet Nam): Analysis of machine learning and deep learning models. *Discov. Artif. Intell.* **5**, 79 (2025).

48. B. P. Q. Nghia, I. Pal, N. Chollacoop, L. H. Nguyen, L. Van Thinh, T. M. Tuan, N. V. Tuong, Integrated approach for drought and saline intrusion severity assessment on the coastal Mekong Delta of Vietnam contextualizing physical change to risk management and policy development. *Prog. Disaster Sci.* **23**, 100338 (2024).

49. X. Cai, Q. Qin, J. Shen, H. A. Michael, M. L. Kirwan, P. A. Raymond, Defending saltwater intrusion: The freshwater pushback. arXiv:2509.11412 [physics.geo-ph] (2025).

50. J. L. Sabo, A. Ruhi, G. W. Holtgrieve, V. Elliott, M. E. Arias, P. B. Ngor, T. A. Räsänen, S. Nam, Designing river flows to improve food security futures in the Lower Mekong Basin. *Science* **358**, eaao1053 (2017).

51. S. Kumar, D. D. Tran, J. Wang, L. Feng, A. D. Switzer, E. Park, “Hotspots of sand mining in Southeast Asian rivers” in *AGU Fall Meeting Abstracts* (2023), vol. 2023, p. GC11E-088.

52. S. Kumar, E. Park, D. D. Tran, J. Wang, A. D. Switzer, C. R. Hackney, “Sustainable Riverbed Sand Harvesting (SSH): A novel solution to balance environmental impacts and developmental needs” in *AGU Fall Meeting Abstracts* (2024), vol. 2024, p. SY53E-06.

53. A. C. Alonso, R. van Weerdenburg, B. van Maren, Y. Huismans, L. Stuur, “Modelling sand-mud interaction in Delft3D” (Tech. Rep. 11205286-010-ZWS-0001, Deltares, 2020); https://publications.deltares.nl/11205286_010_0001.pdf.

54. G. D. Egbert, S. Y. Erofeeva, Efficient inverse modeling of barotropic ocean tides. *J. Atmos. Oceanic Tech.* **19**, 183–204 (2002).

55. GEBCO Compilation Group, GEBCO 2019 Grid (British Oceanographic Data Centre, National Oceanography Centre, NERC, UK, 2019); doi:10.5285/836f016a-33be-6ddc-e053-6c86abc0788e.

56. Mekong River Commission, MRC Data and Information Services (MekongInfo Portal); https://portal.mrcmekong.org/home.

57. L. C. van Rijn, Unified view of sediment transport by currents and waves. I: Initiation of motion, bed roughness, and bed-load transport. *J. Hydraul. Eng.* **133**, 649–667 (2007).

58. T. X. Hai, V. V. Nghi, V. H. Hung, D. N. Tuan, D. T. Lam, C. T. Van, Assessing and forecasting saline intrusion in the Vietnamese Mekong Delta under the impact of upstream flow and sea level rise. *J. Environ. Sci. Eng. B* **8**, 174–185 (2019).

59. W. Mueller, D. Zamrsky, G. O. Essink, L. E. Fleming, A. Deshpande, K. C. Makris, B. W. Wheeler, J. N. Newton, K. M. V. Narayan, A. M. Naser, M. O. Gribble, Saltwater intrusion and human health risks for coastal populations under 2050 climate scenarios. *Sci. Rep.* **14**, 15881 (2024).

60. T. T. Nguyen, K. Stattegger, D. Unverricht, C. Nittrouer, P. V. Phach, P. Liu, D. DeMaster, B. V. Dung, L. D. Anh, M. D. Dong, Surface sediment grain-size distribution and sediment transport in the subaqueous Mekong Delta, Vietnam. *Vietnam J. Earth Sci.* **39**, 193–209 (2017).

61. J. D. Stephens, M. A. Allison, D. R. D. Leonardo, H. D. Weathers, A. S. Ogston, R. L. McLachlan, F. Xing, E. A. Meselhe, Sand dynamics in the Mekong River channel and export to the coastal ocean. *Cont. Shelf Res.* **147**, 38–50 (2017).

62. A. S. Ogston, M. A. Allison, J. C. Mullarney, C. A. Nittrouer, Sediment- and hydro-dynamics of the Mekong Delta: From tidal river to continental shelf. *Cont. Shelf Res.* **147**, 1–6 (2017).

63. G. M. Kondolf, Y. Gao, G. W. Annandale, G. L. Morris, E. Jiang, J. Zhang, Y. Cao, P. Carling, K. Fu, Q. Guo, R. Hotchkiss, C. Peteuil, T. Sumi, H. W. Wang, Z. Wang, Z. Wei, B. Wu, C. Wu, C. T. Yang, Sustainable sediment management in reservoirs and regulated rivers: Experiences from five continents. *Earth's Future* **2**, 256–280 (2014).

64. T. K. O. Ta, V. L. Nguyen, M. Tateishi, I. Kobayashi, S. Tanabe, Y. Saito, Holocene delta evolution and sediment discharge of the Mekong River, southern Vietnam. *Quat. Sci. Rev.* **21**, 1807–1819 (2002).

65. T. Tamura, K. Horaguchi, Y. Saito, V. L. Nguyen, M. Tateishi, T. K. O. Ta, F. Nanayama, K. Watanabe, Monsoon-influenced variations in morphology and sediment of a mesotidal beach on the Mekong River delta coast. *Geomorphology* **116**, 11–23 (2010).

66. T. T. Nguyen, D. H. Cuong, K. Stattegger, B. V. Dung, S. Yang, N. T. K. Chi, N. X. Tung, N. V. Tinh, N. T. Nga, Depositional sequences of the Mekong river delta and adjacent shelf over the past 140 kyr, Southern Vietnam. *J. Asian Earth Sci.* **206**, 104634 (2021).

67. M. Gugliotta, Y. Saito, T. K. O. Ta, V. L. Nguyen, T. Tamura, Z. Wang, A. D. L. Croix, R. Nakashima, Abandonment and rapid infilling of a tide-dominated distributary channel at 0.7 ka in the Mekong River Delta. *Sci. Rep.* **11**, 11040 (2021).

68. M. A. Allison, C. A. Nittrouer, A. S. Ogston, J. C. Mullarney, T. T. Nguyen, Sedimentation and survival of the Mekong Delta: A case study of decreased sediment supply and accelerating rates of relative sea level rise. *Oceanography* **30**, 98–109 (2017).

69. G. Godin, *The Analysis of Tides* (University of Toronto Press, 1972).
